# Variations in Substance Use Prevalence Estimates and Need for Interventions among Adult Emergency Department Patients Based on Different Screening Strategies Using the ASSIST

**DOI:** 10.5811/westjem.2016.3.29723

**Published:** 2016-05-10

**Authors:** Roland C. Merchant, Tao Liu, Janette R. Baird

**Affiliations:** * Brown University, Alpert Medical School, Department of Emergency Medicine, Providence, Rhode Island; † Brown University, School of Public Health, Department of Epidemiology, Providence, Rhode Island; ‡ Brown University, School of Public Health, Center for Statistical Sciences, Department of Biostatistics, Providence, Rhode Island

## Abstract

**Introduction:**

Among adult emergency department (ED) patients, we sought to examine how estimates of substance use prevalence and the need for interventions can differ, based on the type of screening and assessment strategies employed.

**Methods:**

We estimated the prevalence of substance use and the need for interventions using the Alcohol, Smoking and Substance Involvement Screening Test (ASSIST) in a secondary analysis of data from two cross-sectional studies using random samples of English- or Spanish-speaking 18–64-year-old ED patients. In addition, the test performance characteristics of three simplified screening strategies consisting of selected questions from the ASSIST (lifetime use, past three-month use, and past three-month frequency of use) to identify patients in need of a possible intervention were compared against using the full ASSIST.

**Results:**

Of 6,432 adult ED patients, the median age was 37 years-old, 56.6% were female, and 61.6% were white. Estimated substance use prevalence among this population differed by how it was measured (lifetime use, past three-month use, past three-month frequency of use, or need for interventions). As compared to using the full ASSIST, the predictive value and accuracy to identify patients in need of any intervention was best for a simplified strategy asking about past three-month substance use. A strategy asking about daily/near-daily use was better in identifying patients needing intensive interventions. However, some patients needing interventions were missed when using these simplified strategies.

**Conclusion:**

Substance use prevalence estimates and identification of ED patients needing interventions differ by screening strategies used. EDs should carefully select strategies to identify patients in need of substance use interventions.

## INTRODUCTION

Recent research indicates high prevalences of alcohol, smoking and drug use among United States (U.S.) emergency department (ED) adult patients.^1^–^6^ However, estimated ED patient substance-use prevalence and anticipated need for interventions likely are impacted by the screening strategies used to measure them, such as who is screened; where, when, how and by whom screening is conducted; the simplicity or complexity of these strategies; the types of screening instruments used; and the domains these instruments measure. Variability across screening strategies may lead to disparate or contradictory recommendations for addressing substance use among ED patients, which in turn could have consequent deleterious effects on ED and public health priorities chosen for substance use prevention, treatment efforts and the allocation of extramural funding to evaluate interventions.

Although ED-based screening and initiation of consequent interventions are recommended for alcohol and smoking,^7^–^9^ this practice is not yet routine.^10^–^18^ Comprehensive assessments of substance use and intervention need can be a time-consuming process, which can discourage their use. An enticing way to assess ED patients substance use is to use simplified screening-question strategies involving one or two screening questions that might identify individuals in possible need of an intervention (e.g., “In the past 3 months, did you drink alcohol?”).^3^,^4^ This strategy might be applied by ED staff at triage or incorporated into the electronic medical record (EMR), and the results could prompt or obviate the need for an intervention during the clinical care encounter. Before such simplified strategies can be recommended, their yield and accuracy against relatively more comprehensive screening and assessment strategies need to be evaluated.

Our primary aim was to examine how estimates about the prevalence of substance use among adult ED patients differ when these estimates are based on the following: any lifetime, any past three-month, or past three-month frequency of substance use; or according to the need for any, a brief, or an intensive intervention per the Alcohol, Smoking and Substance Involvement Screening Test (ASSIST).^19^ Our secondary goal was to compare the ability of three simplified screening-question strategies to identify adult ED patients in need of any intervention vs. no intervention, and in need of an intensive intervention vs. no intensive intervention (i.e., no intervention needed or only a brief intervention [BI] needed), to the full ASSIST as the “gold standard.” The three simplified strategies constituted screening based on any lifetime use, any past three-month use, or past three-month frequency of use.

## METHODS

### Study design and setting

This investigation was a secondary analysis of two concurrent studies on substance use at two EDs affiliated with a medical school in the same hospital system and city from July 2010 to December 2012. The data were based on two cross-sectional studies that involved surveying random samples of adult ED patients. The hospital institutional review board approved the study.

### Selection of participants

For both studies, bilingual (English- and Spanish-speaking) research assistants (RAs) randomly selected ED patients for possible study inclusion, reviewed their EMR for exclusion criteria, and confirmed study eligibility through a brief interview. A random sample of patients present in the ED during study collection periods was approached and evaluated for study inclusion through random selection to their patient care rooms. If the ED EMR indicated that a patient potentially was study eligible, a RA would confirm study eligibility through a brief interview. Data collection for the study was performed from 8 AM to midnight seven days/week when bilingual (English- and Spanish-speaking) RAs were available to conduct the study.

Patients were study eligible if they were 18–64 years-old; English- or Spanish-speaking; not critically ill or injured; not prison inmates, under arrest, nor undergoing home confinement; not presenting for an acute psychiatric illness; not requesting treatment for substance use; not intoxicated; and did not have a physical or mental impairment that prevented them from providing consent or participating in the study. The study population aimed to reflect the general adult ED population that would be included in a screening, brief intervention, and referral to treatment (SBIRT) program, i.e., excluding those presenting for evaluation of their substance use, those acutely intoxicated, and those undergoing a formal substance use or psychiatric evaluation.

### Methods and measurements

Participants completed the ASSIST, which we adapted for these studies. (See supplemental material for an English-language copy of the study instrument.)^19^ The cognitive-based assessments, pilot testing and evaluations of the adapted ASSIST have been described in detail previously.^20^ In brief, this adaption involved preparing it for audio computer self-administered interviewing (ACASI) to increase veracity of responses of sensitive or stigmatizing information (i.e., substance use/misuse)^21^–^25^ and improve the flow of the instrument; clarifying questions, responses, and instructions; distinguishing misuse from the use of prescription drugs; and adding or expanding drug categories (e.g., barbiturates, benzodiazepines, prescription opioid analgesics). Cronbach’s α ranged from 0.86 to 0.95 for the drug categories assessed in our adapted ASSIST.

**Figure 1:**
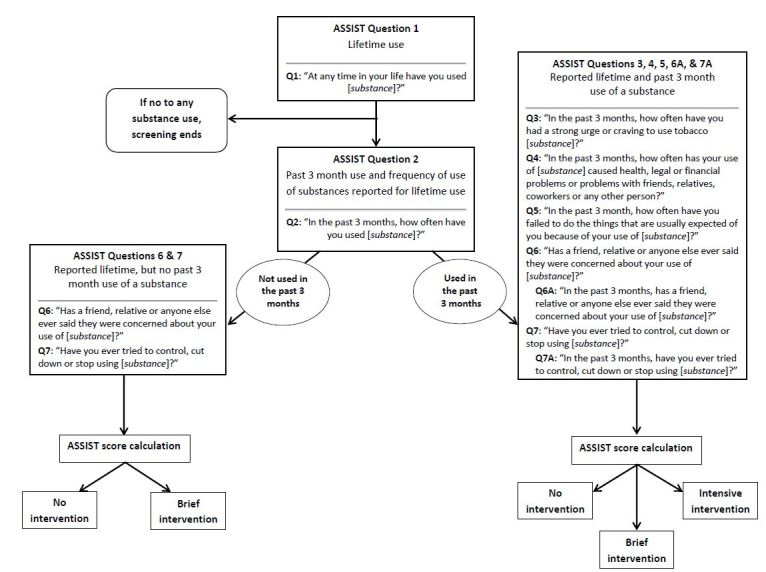
ASSIST screening and substance misuse intervention algorithm. *ASSIST*, Alcohol, Smoking and Substance Involvement Screening Test

As shown in Figure 1, participants first were asked in the ASSIST about any lifetime substance use by substance category, and if they indicated lifetime use of a given substance, they were asked about any past three-month use and frequency of use during the past three months (ASSIST Questions 1 and 2). Following these initial questions, the ASSIST proceeded through an evaluation of substance-use severity. Substance-specific scores are calculated for those who have used a given substance; if an individual has not used that substance, no score is calculated. Per World Health Organization (WHO) recommendations, an ASSIST score of ≥4 points for smoking or any drug category or a score of ≥11 points for alcohol suggests a need for BI, and a score of ≥27 points for any substance suggests a need for a more intensive intervention.^19^ Those who report use in the past three months are assessed for the need of a BI or an intensive intervention, while those who report ever using a given substance (lifetime use) but deny past 3-month use are assessed for the need for a BI only.

In addition to the ASSIST, we queried participants about the specific drugs that they had used within the past three months and whether or not these drugs had been injected or prescribed. (See supplemental material [Appendix A].) The reading level of study questionnaires in English was at a Flesch-Kincaid grade level of 6.6 (Microsoft Word; Microsoft Corp., Redmond, WA) and in Spanish was at a Huerta Reading Ease score of 80, both indicating an easy reading level.^26^ Participants completed the questionnaires in approximately 10–15 minutes.

The RAs received 40 hours of training on the study protocol, including mock interviews with the study investigators and pilot testing of the study protocol prior to collecting data for the study. The RAs met with the study investigators throughout the study to discuss procedural issues arising from the conduct of the study. To ensure fidelity to the study protocol, study investigators directly observed RAs during participant encounters. Deviations from the study protocol were addressed and suggestions for improvement were provided.

### Analysis

Analysis of the study and presentation of study findings followed current Strengthening the Reporting of Observational Studies in Epidemiology (STROBE) recommendations for cross-sectional studies (www.strobe-statement.org). We summarized study eligibility assessments and enrollment using current recommendations,^27^ participant demographic characteristics, and responses to the ASSIST. ASSIST scores were calculated for each participant. The need for any, a brief, or more intensive intervention was calculated according to WHO recommendations for all participants, and as stratified by those reporting no past three-month or any past three-month use of a given substance.^19^

For the primary aim, substance use prevalence was estimated for each substance use category (e.g., smoking, alcohol, benzodiazepines) as stratified by responses to Questions 1 and 2 of the ASSIST (any lifetime use, any past three-month use, and past three-month frequency of use), and by the need for interventions using ASSIST scores. Substance use was ranked in order of decreasing magnitude of prevalence or frequency, respectively, across all substance categories according to these strata. We estimated accompanying 95% confidence intervals (CIs) for the rankings. The ranks were provided to assist in distinguishing differences among proportions within each category.

For the secondary aim, we calculated the test performance characteristics for the ability of the three simplified screening question strategies to identify individuals in need of any intervention vs. no intervention, and in need of an intensive intervention vs. no intensive intervention (i.e., no intervention needed or only a BI needed), as compared to the full ASSIST as the “gold standard.” The three simplified strategies constituted screening based on any lifetime use, any past three-month use, or past three-month frequency of use (per ASSIST Questions 1 and 2). Sensitivity, specificity, negative and positive predictive values, and accuracy with corresponding 95% CIs were estimated. We performed all analyses using STATA 13 (Stata Corporation, College Station, TX).

## RESULTS

### Participant eligibility assessment, enrollment, and demographic characteristics

**Figure 2:**
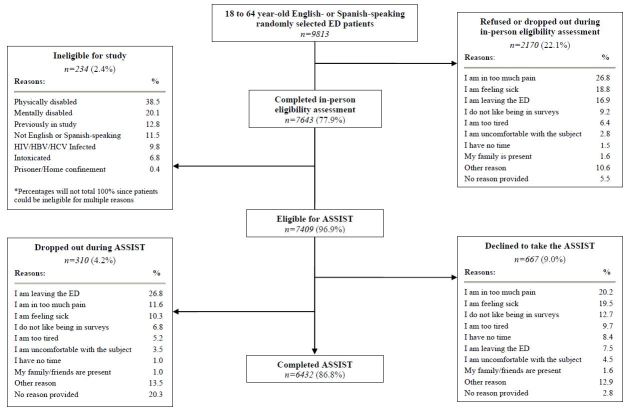
Eligibility assessment and enrollment. *ASSIST*, Alcohol, Smoking and Substance Involvement Screening Test; *HBV*, Hepatitis B Virus; *HCV*, Hepatitis C Virus; *HIV*, human immunodeficiency virus; *ED*, emergency department

Participant eligibility assessment and enrollment results are depicted in Figure 2. As shown, of the 9,813 randomly selected 18- to 64-year-old English- or Spanish-speaking ED patients, 78% (7,643) were assessed in person for study eligibility. Of these, 97% (7,409) were eligible to complete the ASSIST, and 87% (6,432) completed it and comprised the final study sample. The majority of participants were female, white/non-Hispanic, most had 12 or fewer years of formal education, had private healthcare insurance, were married or part of an unmarried couple, were employed, not homeless, and received their medical care from a private clinic/practice (Table 1).

In regards to specific substances used among these ED patients, the highest past three-month use prevalences for illicit drugs were cocaine (4.4%), crack (2.6%), and ecstasy/3,4-methylenedioxy-N-methylamphetamine (MDMA) (1.0%) (Supplemental Table 1). Although among all participants the highest past three-month use prevalences for prescription opioids were acetaminophen and hydrocodone (4.0%), acetaminophen and oxycodone (3.6%), and oxycodone (1.1%), only 2.2% of all participants stated that had been prescribed acetaminophen and hydrocodone, 1.9% acetaminophen and oxycodone, and 0.7% oxycodone within the past three months. Lifetime prevalence of injection-drug use was 4.4% and was 1.7% for past three-month use, predominately heroin (0.93%) and cocaine (0.56%).

### Differences in substance use prevalence estimates among ED patients

When estimating the prevalence of substance use based on lifetime use or past 3-month use, the rank order was the same for the first three substances (alcohol, smoking, and then marijuana) (Table 2). When estimating substance use prevalence according to daily/near-daily use, smoking had the highest rank, followed by marijuana, alcohol, and then prescription opioids. There were subtle differences in the ranks for the remaining drugs across these three ways of estimating the prevalence of substance use.

When estimating substance use prevalence based on the need for interventions (Table 3a and 3b), the need for any intervention or at least a BI among these patients was greatest for smoking, marijuana, and then alcohol; however, the need for an intensive intervention was greatest for smoking, alcohol, and then marijuana. The need for any, a brief, or an intensive intervention differed slightly for the remaining substances.

The relative rank order of the need for a BI also differed when ASSIST score results were stratified by lifetime only vs. past three-month use. Among those who reported lifetime-only use, the need for a BI was greatest for opioids, methadone or buprenorphine, prescription opioid analgesics, barbiturates and then cocaine or crack. However, among those who reported past three-month substance use, the need for a BI was greatest for smoking, marijuana, amphetamines, prescription opioid analgesics, and then methamphetamines. The need for an intensive intervention based on ASSIST scores among those who reported past three-month substance use was greatest for opioids, gamma hydroxybutyrate (GHB), methadone or buprenorphine, cocaine or crack, and then barbiturates. The relative need for an intervention differed for specific substances when comparing past or no past three-month use of these substances. For example, the need for a BI for illicit opioids was greater among those who reported no past three-month use of this substance; whereas for smoking, the need for a BI was greater for those who reported smoking in the past three months.

### Performance of simplified screening question strategies in identifying need for substance use interventions

The performance of using simplified screening question strategies (any lifetime use, any past three-month use, or daily/near-daily use) as compared to the full ASSIST as the “gold standard” to identify need for WHO-recommended substance use interventions is shown in Table 4a–4d. Across all substances, sensitivity decreased while specificity increased when using querying about lifetime use vs. past three-month vs. daily/near-daily use. Also across substances, querying about past three-month use generally performed better in regards to predictive value and accuracy for any intervention need, but querying about daily/near-daily use was generally better for identifying intensive intervention need.

The test performance characteristics of these three simplified screening strategies varied by substance category. For any smoking intervention, predictive values and accuracy of querying about past three-month use was higher than screening for lifetime alone or frequency of use (daily/near-daily use). However, frequency of use performed better for identifying need for smoking intensive interventions, although the positive predictive value was low. For any or alcohol intensive interventions, querying about frequency of use performed better than the other screening strategies. For any marijuana interventions, querying about past three-month use performed better, but querying about frequency of use was better for intensive interventions. For all other drugs, querying about past three-month use was better than other queries for any intervention need, yet querying about frequency of use was better for intensive intervention need.

## DISCUSSION

Our investigation demonstrates how estimated substance use prevalence among ED patients can differ depending on how it is derived. For example, prescription opioid analgesics is only sixth when estimating its prevalence based on lifetime use, but rises to fourth in prevalence when considering past three-month use, daily/near-daily use, or the need for a BI based on past three-month use. Likewise, smoking becomes the number one substance use problem when considering the need for interventions, rather than measuring lifetime or past three-month use prevalence. Subtle small differences in estimates such as these can have large public health implications on which substances are prioritized for interventions in United States EDs.

This investigation adds a unique perspective to the small number of large sample studies estimating the extent of adult ED patient substance use. The results from these prior studies also illustrate how differences in who is screened, how they are screened, and how prevalence is estimated can change perspectives on the extent of substance use in this setting. Wu, et al. conducted a secondary analysis of past year prevalence of alcohol and drug use disorders using ACASI-collected data from persons ≥18 years-old in the U.S. who participated in the 2007–2009 National Survey on Drug Use and Health and who had visited an ED at least once within the past year.^2^ Approximately 66% of those who had visited an ED drank alcohol, 12% had used at least one of nine different drugs within the past year, 10% met criteria for alcohol abuse or dependence and 4% for drug abuse or dependence per Diagnostic and Statistical Manual of Mental Disorders (DSM-IV) criteria. Wu, et al. observed that the prevalence of substance use disorders was greatest for heroin, sedatives, cocaine, and then opioids. As noted, Wu, et al. reported a lower prevalence of drug use and markedly different order of drugs of concern than for our study. These differences likely are due to the study methodology, drug categories used, their inability to measure recency of substance use, and use of DSM-IV criteria for abuse and dependence.

Four recent U.S. ED-based studies screened patients concurrently with their ED visit, as we did in our investigation. Two employed a brief screening strategy administered aloud by nurses at ED triage. From 2009 to 2012 among ≥18-year-old ED patients in Macon, Georgia, Johnson, et al. evaluated three one-question screening tools for past year tobacco product use, alcohol use (≥4 drinks in a day for women, ≥5 drinks in a day for men), and drug use (“pot [marijuana], use of another street drug, or use of a prescription painkiller, stimulant or sedative for a non-medical reason”), which had been adapted from primary care settings.^3^ Approximately 22% screened positive for at-risk alcohol or drug use. Hankin, et al. from 2009 to 2010 employed a screening approach similar to that by Johnson, et al. among a predominately Black, non-Hispanic population at the Grady Hospital ED in Atlanta, Georgia.^4^ Patients with a positive screen were administered the ASSIST through an interview with a health educator. Of those screened, 27.6% had a positive one-question screen for alcohol or drug use within the past year; marijuana and cocaine were the most commonly used drugs. The lower yields of screening for these studies as compared to our study might be due to the type of screening instruments used and mode of screening (nurse-administered aloud at ED triage). Employing three different screening instruments at EDs in six states among ≥18-year-old English-speaking patients, Sanjuan, et al.^5^ and Konstantopoulos, et al.^6^ found that 39% reported daily tobacco use, 45% risky alcohol use, 22% any past 30-day drug use, and 17% moderate to severe drug problems. The most frequently reported drugs used were marijuana, cocaine, “street opioids” and prescription opioids. Although Spanish-speaking ED patients were excluded and different instruments were used, the prevalence of use from these two analyses is relatively similar to ours.

In their practical application, our study findings also indicate how screening strategies can affect adult ED patients identified (or missed) as possibly needing an intervention. For example, a simplified screening question strategy that queries about past three-month use to identify adult ED patients needing any intervention would perform well for smoking, marijuana, and other drugs, but would perform poorly for alcohol. Likewise, querying about daily/near-daily use to identify need for an intensive intervention would perform well for marijuana, other drugs, and alcohol, but less well for smoking.

As the ASSIST assessments that stratify by lifetime or past three-month use remind us, remarkably high proportions of ED patients who deny current or recent substance use still qualify for a BI per WHO recommendations. This finding indicates that asking about lifetime use and not just past three-month or frequency of use remains an important facet in identifying those who might benefit from substance use interventions. This stratification also demonstrates that the relative need for an intervention can vary substantially when considering past three-month use, as the example of illicit opioids and smoking illustrate. One implication for ED clinical practice is to recognize that a need for an intervention is based on more than current substance use. Querying about lifetime and current use and frequency of use can help to identify those who need assistance. The challenge for the future is to learn how to screen efficiently yet effectively in EDs to identify those who might benefit from assistance because of their substance use. Unfortunately, we do not yet have a single perfect instrument to screen adult ED patients quickly and easily for substance abuse in a manner that identifies all of those in need of an intervention, without overburdening providers and patients, and minimizes false positive and negative results.

One caveat is that effective interventions for adult ED patients to help them reduce or eliminate their substance use continue to elude us. Although BIs have been recommended^28^ for alcohol among adolescent and adult ED patients, studies evaluating them have had mixed results.^29^–^42^ Three different recently published studies also observed the following: (1) no short-term (three-month follow-up) benefit from a BI in reducing drug use or increasing uptake of drug treatment services;^43^ (2) no differences in past 30-day drug use abstinence six months post-enrollment^44^; and no advantage to a BI with telephone boosters as compared with screening, assessment and referral to treatment or minimal screening only in terms of drug use at three, six, and 12 months post-enrollment.^45^ As such, although instruments such as the ASSIST might indicate the need for an intervention, the optimal type of intervention needed is not yet known.

## LIMITATIONS

This study had several limitations. Although care was taken to reach a representative sample of adult patients at these two EDs not presenting for a substance use or psychiatric evaluation, those who were excluded might have different substance use profiles and need for interventions. An advantage of this study is that random sampling of patients was used and Spanish-speaking patients were included, which should increase the internal and external validity of the study findings. Of course, patients at EDs in other locales might have a different spectrum of substance use. Although we provide estimates of predictive value and accuracy for the simplified screening strategies, such estimates are based on the prevalence at these EDs, and likely would be different at other EDs. Nevertheless, these parameters provide an illustration of the application of the screening strategies. Even though confidentiality and fidelity of responses likely was enhanced by the use of the ACASI approach,^21^–^25^ we cannot be assured that all participants answered the questions truthfully. As would be expected, other screening strategies and substance-use evaluation instruments other than the ASSIST might yield different results. In addition, because we only used questions taken from the ASSIST and not any other instrument, we cannot determine how the three simplified screening strategies would compare to other screening instruments. Comparisons to different instruments might have led to different conclusions.

## CONCLUSION

The results of this investigation indicate that estimated substance use prevalence among adult ED patients differs according to how it is measured. In addition, the yield from simplified screening question strategies can vary by substance, and risk missing patients who might benefit from an intervention. Public policy makers and EDs contemplating substance use screening programs should be cognizant that the priorities for what substances need intervention and the types of interventions needed are highly dependent on how adult ED patients are assessed for substance use.

## Supplementary Information



## Figures and Tables

**Table 1:** Demographic characteristics of participants.

Demographic characteristics	n=6432
Median age, years (IQR)	37 (26–48) %

Gender	
Female	56.6
Male	43.4
Ethnicity/race	
White, non-Hispanic	61.6
White, Hispanic	11.2
Black/African-American, non-Hispanic	17.3
Black/African-American, Hispanic	6.5
Other	3.3
Years of formal education	
<12 years	25.6
Grade 12	29.8
College 1–3 years	26.9
College 4 years (college graduate)/ >College	17.6
Health insurance status	
Private	40.8
Governmental	34.0
None	25.1
Don’t know/refuse to answer	0.1
Partner status	
Married	27.9
Divorced/widowed/separated	18.0
Never married	38.8
Unmarried couple	15.4
Homeless status	
Currently homeless	5.5
Past 12 months homeless	3.1
Never/not homeless past 12 months	91.4
Employment status	
Employed	48.8
Disability	18.0
Student	8.3
Unemployed	24.8
Don’t know/refuse to answer	0.1
Usual source of medical care	
Private clinic/practice	46.4
Hospital or community health clinics	26.2
Emergency department	24.8
Urgent care center	2.5
Don’t know/refuse to answer	0.2

*IQR*, interquartile range

**Table 2:** Extent of substance use as assessed by any lifetime use, any past 3-month use, or past 3-month frequency of use (n=6432).

	Any lifetime or any past 3-month use					Past 3-month frequency of use					
	
	Never used or misused	Lifetime use		Past 3-month use		5 to 7 days a week		1 to 4 days a week	1 to 2 days a month	1 to 2 days in the past 3 months	Not used in the past 3 months
	
	%	%	Rank of % (95% CI)	%	Rank of % (95% CI)	%	Rank of % (95% CI)	%	%	%	%
Smoking	33.7	66.3	2 (2,2)	43.7	2 (2,2)	32.8	1 (1,1)	5.3	2.5	3.1	56.1
Alcoholic beverages	13.1	86.9	1 (1,1)	61.6	1 (1,1)	6.5	3 (2,3)	19.3	18.5	17.3	38.0
Marijuana	39.8	60.2	3 (3,3)	26.6	3 (3,3)	9.5	2 (2,2)	6.9	4.5	5.7	72.9
Cocaine or crack	75.5	24.5	4 (4,4)	5.8	5 (4,5)	1.0	5 (4,6)	1.0	1.7	2.2	93.7
Methamphetamines	90.1	9.9	7 (7,8)	1.6	9 (8,10)	0.2	9 (9,12)	0.2	0.4	0.8	98.0
Inhalants	94.5	5.5	11 (10,11)	0.6	12 (12,12)	0.1	11 (9,13)	0.1	0.1	0.3	99.1
Hallucinogens	84.0	16.0	5 (5,5)	1.2	11 (10,11)	0.1	12 (9,12)	0.1	0.3	0.8	98.1
Illicit opioids	91.6	8.4	9 (9,9)	2.3	7 (7,7)	0.7	6 (6,7)	0.6	0.4	0.6	97.2
Gamma-hydroxybutyrate (GHB)	98.4	1.6	13 (13,14)	0.2	14 (13,14)	0.1	13 (12,14)	0.0	0.0	0.1	99.1
Amphetamines	93.8	6.2	10 (10,11)	1.7	8 (8,9)	0.2	10 (9,13)	0.4	0.4	0.7	97.6
Benzodiazepines	90.3	9.7	8 (7,8)	3.6	6 (6,6)	0.7	7 (5,7)	0.9	0.8	1.2	95.9
Barbiturates	98.5	1.5	14 (13,14)	0.3	13 (13,14)	0.1	14 (12,14)	0.1	0.0	0.1	99.2
Methadone or buprenorphine	96.8	3.2	12 (12,12)	1.3	10 (9,11)	0.3	8 (8,9)	0.3	0.3	0.3	97.9
Prescription opioid analgesics	85.1	14.9	6 (6,6)	6.3	4 (4,5)	1.2	4 (4,5)	1.8	1.6	1.7	93.1

*n.b.* Rows do not total to 100% because participants could refuse to answer.

**Table 3a:** Extent of substance use according to the need for substance misuse interventions per WHO recommendations based on ASSIST scores.

	WHO recommendations for interventions													
	
	Entire population (n=6432)						No past 3-month substance use				Any past 3-month substance use			
	
	Any intervention		Brief intervention		Intensive intervention			Brief intervention			Brief intervention		Intensive intervention	
					
Substances	%	Rank of % (95% CI)	%	Rank of % (95% CI)	%	Rank of % (95% CI)	n	%	Rank of % (95% CI)	n	%	Rank of % (95% CI)	%	Rank of % (95% CI)
Smoking	43.9	1 (1,1)	34.8	1 (1,1)	9.1	1 (1,1)	1449	10.4	7 (5,8)	2812	74.3	1 (1,1)	13.7	11 (6,10)
Alcoholic beverages	16.2	3 (3,3)	11.6	3 (3,3)	4.6	2 (2,2)	1628	0.0	14 (14,14)	3959	18.8	14 (13,14)	7.6	14 (13,14)
Marijuana	22.6	2 (2,2)	19.5	2 (2,2)	3.1	3 (3,3)	2162	3.5	12 (10,12)	1708	69.1	2 (2,3)	11.6	13 (9,13)
Cocaine or crack	7.1	4 (4,5)	5.2	4 (4,5)	1.9	4 (4,5)	1203	12.1	5 (4,7)	372	49.7	8 (6,11)	33.1	4 (2,6)
Methamphetamines	1.6	8 (8,11)	1.4	8 (8,10)	0.2	10 (9,12)	535	5.6	9 (8,12)	101	56.4	5 (3,10)	12.9	12 (9,14)
Inhalants	0.5	12 (12,13)	0.4	13 (12,13)	0.1	12 (10,14)	315	1.6	13 (12,13)	41	46.3	10 (3,13)	19.5	8 (4,13)
Hallucinogens	1.3	11 (9,11)	1.2	10 (8,11)	0.2	11 (9,12)	950	3.7	11 (10,12)	80	48.8	9 (4,12)	13.8	10 (9,14)
Illicit opioids	3.4	7 (6,7)	2.3	7 (6,7)	1.1	6 (6,7)	390	20.8	1 (1,3)	150	42.7	12 (8,13)	48.0	1 (1,3)
GHB	0.2	14(14,14)	0.1	14 (14,14)	0.1	14 (13,4)	93	6.5	8 (5,12)	10	30.0	13 (4,14)	40.0	2 (1,12)
Amphetamines	1.5	10 (8,11)	1.2	9 (8,11)	0.3	9 (9,11)	288	5.6	10 (8,12)	109	56.9	3 (3,10)	15.6	9 (8,13)
Benzodiazepines	3.6	6 (6,7)	2.6	6 (6,7)	1.0	7 (6,7)	394	10.4	6 (4,8)	232	53.0	7 (4,10)	28.0	7 (3,8)
Barbiturates	0.4	13(12,13)	0.3	12 (12,13)	0.1	13(12,14)	80	16.3	4 (1,7)	16	43.8	11 (2,13)	31.3	5 (1,13)
Methadone or buprenorphine	1.5	9 (8,11)	1.1	11 (8,11)	0.5	8 (8,8)	121	19.8	2 (1,4)	82	56.1	6 (3,11)	35.4	3 (2,7)
Prescription opioid analgesics	6.8	5 (4,5)	5.0	5 (4,5)	1.8	5 (4,5)	550	16.4	3 (2,4)	407	56.5	4 (3,8)	29.0	6 (3,7)
Total ASSIST drug score	28.6													
Total ASSIST score	53.8													

*χ̄*, mean; *SE*, standard error; *ASSIST*, Alcohol, Smoking and Substance Involvement Screening Test; *WHO*, World Health Organization; *GHB,* gamma-hydroxybutyrate

**Table 3b:** 

	ASSIST score for reporting any lifetime use	
	
Substances	n	χ̄ (SE)
Smoking	4261	13.3 (0.17)
Alcoholic beverages	5587	6.5 (0.11)
Marijuana	3870	5.9 (0.14)
Cocaine or crack	1575	5.4 (0.24)
Methamphetamines	636	2.6 (0.24)
Inhalants	356	1.9 (0.30)
Hallucinogens	1030	1.4 (0.13)
Illicit opioids	540	7.8 (0.50)
GHB	103	2.5 (0.71)
Amphetamines	397	4.0 (0.37)
Benzodiazepines	626	7.1 (0.43)
Barbiturates	96	4.3 (0.88)
Methadone or buprenorphine	203	9.2 (0.82)
Prescription opioid analgesics	957	8.6 (0.36)
Total ASSIST drug score	4146	13.6 (0.44)
Total ASSIST score	5898	25.3 (0.45)

*χ̄,* mean; *SE*, Standard Error; *ASSIST*, Alcohol, Smoking and Substance Involvement Screening Test; *GHB*, gamma-hydroxybutyrate

**Table 4a:** Test performance characteristics of simplified screening strategies for substance misuse interventions as compared to the full ASSIST.

	Need for any intervention vs. no intervention					

	Marijuana			Any other drug		

	Lifetime use only	Past 3-month use	Daily to near daily use	Lifetime use only	Past 3-month use	Daily to near daily use
	
	% (95% CI)	% (95% CI)	% (95% CI)	% (95% CI)	% (95% CI)	% (95% CI)
	
Sensitivity	100	94.8 (93.6, 95.9)	42.0 (39.4, 44.6)	100	78.2 (75.3, 80.9)	22.5 (19.8, 25.5)
Specificity	51.1 (49.7, 52.5)	93.3 (92.6, 94.0)	100	71.0 (69.8, 72.2)	97.3 (96.9, 97.7)	100
PPV	37.5 (36.0, 39.1)	80.7 (78.7, 82.5)	100	35.3 (33.4,37.2)	82.2 (79.4,84.7)	100
NPV	100	98.4 (98.0, 98.7)	85.4 (84.5, 86.3)	100	96.6 (96.1, 97.0)	89.1 (88.3, 89.9)
Accuracy	62.2 (61.0, 63.4)	93.7 (93.1, 94.3)	86.8 (86.0, 87.6)	75.0 (73.9, 76.1)	94.7 (94.2, 95.3)	89.4 (88.7, 90.2)

*ASSIST*, Alcohol, Smoking and Substance Involvement Screening Test; *PPV*, positive predictive value; *NPV*, negative predictive value

**Table 4b:** 

	Need for any intervention vs. no intervention					

	Smoking			Alcohol		
	
	Lifetime use only	Past 3-month use	Daily to near daily use	Lifetime use only	Past 3-month use	Daily to near daily use
	
	% (95% CI)	% (95% CI)	% (95% CI)	% (95% CI)	% (95% CI)	% (95% CI)
	
Sensitivity	100	94.7 (93.8,95.5)	74.7 (73.1,76.3)	100	100	30.9 (28.1, 33.8)
Specificity	60.0 (58.4,61.6)	96.2 (95.5,96.8)	100	15.3 (14.3, 16.2)	45.6 (44.3, 47.0)	98.2 (97.8, 98.5)
PPV	66.3 (64.9,67.7)	95.1 (94.2,95.9)	100	18.7 (17.7, 19.7)	26.3 (25.0, 27.7)	77.0 (72.7, 81.0)
NPV	100	95.8 (95.1,96.4)	83.4 (82.3,84.5)	100	100	88.0 (87.1, 88.8)
Accuracy	77.6 (79.6,78.6)	95.5 (95.0,96.0)	88.9 (88.1,89.6)	29.1 (27.9, 30.2)	54.5 (53.3, 55.7)	87.2 (86.4, 88.1)

*ASSIST*, Alcohol, Smoking and Substance Involvement Screening Test; *PPV*, positive predictive value; *NPV*, negative predictive value

**Table 4c:** 

	Need for an intensive intervention vs. no intensive intervention (no intervention or a brief intervention)					
	
	Smoking			Alcohol		
	
	Lifetime use only	Past 3-month use	Daily to near daily use	Lifetime use only	Past 3-month use	Daily to near daily use
	
	% (95% CI)	% (95% CI)	% (95% CI)	% (95% CI)	% (95% CI)	% (95% CI)
	
Sensitivity	100	100	94.7 (92.6, 96.4)	100	100	53.8 (48.0, 59.6)
Specificity	37.0 (35.7,38.2)	61.8 (60.6, 63.1)	73.3 (72.2, 74.4)	13.4 (12.6, 14.3)	40.1 (38.8, 41.3)	95.8 (95.3, 96.3)
PPV	13.7 (12.7,14.8)	20.8 (19.3, 22.4)	26.2 (24.4, 28.2)	5.4 (4.8, 6.0)	7.6 (6.7, 8.4)	38.5 (33.8, 43.4)
NPV	100	100	99.3 (99.0, 99.5)	100	100	97.7 (97.3, 98.1)
Accuracy	42.7 (45.1,43.9)	65.3 (64.1, 66.5)	75.2 (74.2, 76.3)	17.4 (16.5, 18.4)	42.8 (41.6, 44.1)	93.8 (93.2, 94.4)

*ASSIST*, Alcohol, Smoking and Substance Involvement Screening Test; *PPV*, positive predictive value; *NPV*, negative predictive value

**Table 4d:** 

	Need for an intensive intervention vs. no intensive intervention (no intervention or a brief intervention)					
	
	Marijuana			Any other drug		
	
	Lifetime use only	Past 3-month use	Daily to near daily use	Lifetime use only	Past 3-month use	Daily to near daily use
	
	% (95% CI)	% (95% CI)	% (95% CI)	% (95% CI)	% (95% CI)	% (95% CI)
	
Sensitivity	100	100	73.2 (66.5, 79.3)	100	100	51.0 (44.7, 57.3)
Specificity	40.7 (39.5, 42.0)	75.6 (74.5, 76.7)	92.5 (91.8, 93.1)	63.9 (62.7, 65.1)	90.7 (89.9, 91.4)	98.9 (98.7, 99.2)
PPV	5.1 (4.4, 5.9)	11.6 (10.1, 13.2)	23.8 (20.4, 27.4)	10.5 (9.3, 11.7)	31.1 (28.0, 34.4)	67.0 (59.9, 73.6)
NPV	100	100	99.1 (98.8, 99.3)	100	100	98.0 (97.6, 98.3)
Accuracy	42.6 (41.4, 43.8)	76.4 (75.3, 77.4)	91.8 (91.2, 92.6)	65.4 (64.2, 66.6)	51.0 (44.7, 57.3)	97.0 (96.6, 97.4)

*ASSIST*, Alcohol, Smoking and Substance Involvement Screening Test; *PPV*, positive predictive value; *NPV*, negative predictive value

## References

[1] Blow FC, Walton MA, Barry KL (2011). Alcohol and drug use among patients presenting to an inner-city emergency department: a latent class analysis. Addict Behav.

[2] Wu LT, Swartz MS, Wu Z (2012). Alcohol and drug use disorders among adults in emergency department settings in the United States. Ann Emerg Med.

[3] Johnson JA, Woychek A, Vaughan D (2013). Screening for at-risk alcohol use and drug use in an emergency department: integration of screening questions into electronic triage forms achieves high screening rates. Ann Emerg Med.

[4] Hankin A, Daugherty M, Bethea A (2013). The Emergency Department as a prevention site: a demographic analysis of substance use among ED patients. Drug Alcohol Depend.

[5] Sanjuan PM, Rice SL, Witkiewitz K (2014). Alcohol, tobacco, and drug use among emergency department patients. Drug Alcohol Depend.

[6] Konstantopoulos WLM, Dreifuss JA, McDermott KA Identifying patients with problematic drug use in the emergency department: results of a multisite study. Annals Emerg Med.

[7] Babcock-Irvin C, Wyer PC, Gerson LW (2000). Preventive care in the emergency department, Part II: Clinical preventive services--an emergency medicine evidence-based review. Society for Academic Emergency Medicine Public Health and Education Task Force Preventive Services Work Group. Acad Emerg Med.

[8] Bernstein SL, Boudreaux ED, Cydulka RK (2006). Tobacco control interventions in the emergency department: a joint statement of emergency medicine organizations. Ann Emerg Med.

[9] American College of Emergency Physicians (2011). Alcohol screening in the emergency department.

[10] Rockett IR, Putnam SL, Jia H (2003). Assessing substance abuse treatment need: a statewide hospital emergency department study. Ann Emerg Med.

[11] Tong EK, Strouse R, Hall J (2010). National survey of U.S. health professionals’ smoking prevalence, cessation practices, and beliefs. Nicotine Tob Res.

[12] Greenberg MR, Weinstock M, Fenimore DG (2008). Emergency department tobacco cessation program: staff participation and intervention success among patients. J Am Osteopath Assoc.

[13] Vokes NI, Bailey JM, Rhodes KV (2006). “Should I give you my smoking lecture now or later?” Characterizing emergency physician smoking discussions and cessation counseling. Ann Emerg Med.

[14] Prochazka A, Koziol-McLain J, Tomlinson D (1995). Smoking cessation counseling by emergency physicians: opinions, knowledge, and training needs. Acad Emerg Med.

[15] Cunningham RM, Harrison SR, McKay MP (2010). National survey of emergency department alcohol screening and intervention practices. Ann Emerg Med.

[16] Yokell MA, Camargo CA, Wang NE (2014). Characteristics of United States Emergency Departments that Routinely Perform Alcohol Risk Screening and Counseling for Patients Presenting with Drinking-related Complaints. West J Emerg Med.

[17] Delgado MK, Acosta CD, Ginde AA (2011). National survey of preventive health services in US emergency departments. Ann Emerg Med.

[18] O’Rourke M, Richardson LD, Wilets I (2006). Alcohol-related problems: emergency physicians’ current practice and attitudes. J Emerg Med.

[19] Humeniuk R, Ali R (2006). Validation of the Alcohol, Smoking and Substance Involvement Screening Test (ASSIST) and pilot brief intervention [electronic resource] : a technical report of phase II findings of the WHO ASSIST Project.

[20] Merchant RC, Baird JR, Liu T (2014). Brief intervention to increase emergency department uptake of combined rapid human immunodeficiency virus and hepatitis C screening among a drug misusing population. Acad Emerg Med.

[21] Perlis TE, Des Jarlais DC, Friedman SR (2004). Audio-computerized self-interviewing versus face-to-face interviewing for research data collection at drug abuse treatment programs. Addiction.

[22] Caldwell DH, Jan G (2012). Computerized assessment facilitates disclosure of sensitive HIV risk behaviors among African Americans entering substance abuse treatment. Am J Drug Alcohol Abuse.

[23] Islam MM, Topp L, Conigrave KM (2012). The reliability of sensitive information provided by injecting drug users in a clinical setting: clinician-administered versus audio computer-assisted self-interviewing (ACASI). AIDS Care.

[24] Newman JC, Des Jarlais DC, Turner CF (2002). The differential effects of face-to-face and computer interview modes. Am J Public Health.

[25] Turner CF, Villarroel MA, Rogers SM (2005). Reducing bias in telephone survey estimates of the prevalence of drug use: a randomized trial of telephone audio-CASI. Addiction.

[26] Fernandez Huerta JM (1959). Medidas sencillas de lecturabilidad (Simple readability measures). Consigna.

[27] von Elm E, Altman DG, Egger M (2008). The Strengthening the Reporting of Observational Studies in Epidemiology (STROBE) statement: guidelines for reporting observational studies. J Clin Epidemiol.

[28] Cunningham RM, Bernstein SL, Walton M (2009). Alcohol, tobacco, and other drugs: future directions for screening and intervention in the emergency department. Acad Emerg Med.

[29] Nilsen P, Baird J, Mello MJ (2008). A systematic review of emergency care brief alcohol interventions for injury patients. J Subst Abuse Treat.

[30] Havard A, Shakeshaft A, Sanson-Fisher R (2008). Systematic review and meta-analyses of strategies targeting alcohol problems in emergency departments: interventions reduce alcohol-related injuries. Addiction.

[31] Yuma-Guerrero PJ, Lawson KA, Velasquez MM (2012). Screening, brief intervention, and referral for alcohol use in adolescents: a systematic review. Pediatrics.

[32] Cochran G, Field C, Caetano R (2014). Injury-related consequences of alcohol misuse among injured patients who received screening and brief intervention for alcohol: a latent class analysis. Substance abuse.

[33] Dent AW, Weiland TJ, Phillips GA (2008). Opportunistic screening and clinician-delivered brief intervention for high-risk alcohol use among emergency department attendees: a randomized controlled trial. Emerg Med Australasia.

[34] Wojnar M, Jakubczyk A (2014). Brief interventions for hazardous and harmful alcohol consumption in accident and emergency departments. Front Psychiatry.

[35] D’Onofrio G, Degutis LC (2002). Preventive care in the emergency department: screening and brief intervention for alcohol problems in the emergency department: a systematic review. Acad Emerg Med.

[36] Taggart IH, Ranney ML, Howland J (2013). A systematic review of emergency department interventions for college drinkers. J Emerg Med.

[37] D’Onofrio G, Fiellin DA, Pantalon MV (2012). A brief intervention reduces hazardous and harmful drinking in emergency department patients. Ann Emerg Med.

[38] D’Onofrio G, Pantalon MV, Degutis LC (2008). Brief intervention for hazardous and harmful drinkers in the emergency department. Ann Emerg Med.

[39] Sommers MS, Lyons MS, Fargo JD (2013). Emergency department-based brief intervention to reduce risky driving and hazardous/harmful drinking in young adults: a randomized controlled trial. Alcohol Clin Exp Res.

[40] Bernstein J, Heeren T, Edward E (2010). A brief motivational interview in a pediatric emergency department, plus 10-day telephone follow-up, increases attempts to quit drinking among youth and young adults who screen positive for problematic drinking. Acad Emerg Med.

[41] Academic ED SBIRT Research Collaborative (2010). The impact of screening, brief intervention and referral for treatment in emergency department patients’ alcohol use: a 3-, 6- and 12-month follow-up. Alcohol Alcohol.

[42] Newton AS, Dong K, Mabood N (2013). Brief emergency department interventions for youth who use alcohol and other drugs: a systematic review. Pediatric emerg care.

[43] Merchant RC, Baird JR, Liu T (2015). Short-term efficacy of a brief intervention to reduce drug misuse and increase drug treatment utilization among adult emergency department patients. Acad Emerg Med.

[44] Woodruff SI, Clapp JD, Eisenberg K (2014). Randomized clinical trial of the effects of screening and brief intervention for illicit drug use: the Life Shift/Shift Gears study. Addict Sci Clin Pract.

[45] Bogenschutz MP, Donovan DM, Mandler RN (2014). Brief intervention for patients with problematic drug use presenting in emergency departments: a randomized clinical trial. JAMA.

