# Supplementary material for: Variations in Substance Use Prevalence Estimates and Need for Interventions among Adult Emergency Department Patients Based on Different Screening Strategies Using the ASSIST
Source: West J Emerg Med. 2016 May 10;17(3):302–14. doi: 10.5811/westjem.2016.3.29723 (PMC4899062; doi:10.5811/westjem.2016.3.29723)
Supplement: Supplementary file 1 [file wjem-17-302-s001.pdf]

<u>ASSIST questionnaire</u>

We will first ask you to complete a brief survey about your use of alcohol, tobacco and drugs. **Even if you do not use alcohol, tobacco and drugs, we are still interested in the information you can provide us.** We will not ask for your name or any other identifying information.

The brief survey will take about 10 minutes to complete.

Based upon the answers you provide, you might be asked to participate in a second part of our study. If you are eligible to participate, we will tell you more about the second part of the study.

Answering our brief survey will not affect the medical care that you receive today. Your participation is voluntary and you have the right to refuse to answer any or all questions.

If you have any questions or concerns about this study, please feel free to contact XXX at XXX.

If you have any questions about your rights as a research subject, please contact the Office of Research Administration Manager, XXX at XXX.

Thank you for your time!

Verbal consent/Accepted?
1 Yes
0 No
99 Research Assistant Only

Verbal consent/Accepted?
1 Yes
0 No
99 Research Assistant Only

Begin Showing Participant How To Use the Computer

Do you have a pet?
1 Yes
0 No
97 Don't know
98 Refuse to answer
99 Staff Only

We will now ask you questions about your use of tobacco, alcohol and drugs at any time in your life and also about your use in the past 3 months.
We will ask about the types of drugs you might have used. For example, prescription pain killers are a type of drug. Examples of prescription pain killers are: OxyContin and Vicodin.
Please hit the button marked "next question" to begin the survey.

1a. At any time in your life, have you used **tobacco** (For example: cigarettes, cigars, or chewing tobacco)?
3 Yes
0 No
97 Don't know
98 Refuse to answer
99 Staff Only

1b. At any time in your life, have you used **alcohol**?
3 Yes
0 No
97 Don't know
98 Refuse to answer
99 Staff Only

1c. At any time in your life, have you used **marijuana**?
3 Yes
0 No
97 Don't know
98 Refuse to answer
99 Staff Only

1d. At any time in your life, have you used **cocaine** (For example: coke, crack)?
3 Yes
0 No
97 Don't know
98 Refuse to answer
99 Staff Only

1e. At any time in your life, have you used **methamphetamines** (For example: *crank*, crystal meth, ecstasy or MDMA, *tweak*)?
3 Yes
0 No
97 Don't know
98 Refuse to answer
99 Staff Only

1f. At any time in your life have you used **inhalants** (For example: gasoline; glues; paint thinners; *poppers*, snappers or nitrous oxide, spray paint or other aerosol; *whippets*)?

Please **DO NOT** count the medications you take for asthma, emphysema, bronchitis and COPD.

3 Yes
0 No
97 Don't know
98 Refuse to answer
99 Staff Only

1g. At <u>any time</u> in your life have you used **hallucinogens** or "trip drugs" (For example: dextromethorphan, *DM* or *DXM*; coricidin C or *Robo*; herbal hallucinogens; ketamine, *K* or *special K*; LSD or *acid*; mushrooms; PCP or *angel dust*; peyote)?

3 Yes
0 No
97 Don't know
98 Refuse to answer
99 Staff Only

1h. At <u>any time</u> in your life have you used **heroin** or **opium**?

3 Yes
0 No
97 Don't know
98 Refuse to answer
99 Staff Only

1i. At <u>any time</u> in your life have you used **GHB** or **GBH** (*G, Georgia Home Boy, Grievous Bodily Harm*)?

3 Yes
0 No
97 Don't know
98 Refuse to answer
99 Staff Only

<u>Information to respondent:</u> ***Sometimes people <u>misuse</u> prescription drugs. People <u>misuse</u> prescription drugs when they:***

- ***Take prescription drugs at higher amounts or more often than they were prescribed***
- ***Take prescription drugs to get high or to avoid withdrawal symptoms***
- ***Take prescription drugs that were NOT prescribed to them***
-

We will now ask about the <u>types</u> of prescription drugs you might have <u>misused</u>.

Please hit "next question"

1m. At <u>any time</u> in your life, have you **misused** amphetamines (For example: Adderall; Concerta; Dexedrine; Focalin; Ritalin)?

3 Yes
0 No
97 Don’t know
98 Refuse to answer
99 Staff Only

1n. At <u>any time</u> in your life have you **misused** benzodiazepines or "benzos" (For example: Ativan; Klonopin; Resotoril; Valium; Xanax)?
3 Yes
0 No
97 Don’t know
98 Refuse to answer
99 Staff Only

1o. At <u>any time</u> in your life have you **misused** barbiturates (For example: Butisol Sodium; Nembutal Sodium; Seconal Sodium; Solfoton?
3 Yes
0 No
97 Don’t know
98 Refuse to answer
99 Staff Only

1p. At <u>any time</u> in your life have you **misused** methadone or Suboxone (buprenorphine)?
3 Yes
0 No
97 Don’t know
98 Refuse to answer
99 Staff Only

1q. At <u>any time</u> in your life have you **misused** prescription pain killers (For example: Darvocet; Demerol; Dilaudid; Fentora; Lortab; Oxycontin; Percocet; Vicodin)?
3 Yes
0 No
97 Don’t know
98 Refuse to answer
99 Staff Only

<u>Information to respondent:</u> ***We will now ask you if you used <u>any</u> <u>type</u> of tobacco, alcohol or drugs in the past 3 months:***

- ***5 to 7 days a week***
- ***1 to 4 days a week***
- ***1 to 2 days a month***
- ***1 to 2 days in the past 3 months***
- ***Not used in the past 3 months***

Please hit "next question"

[If yes to 1a]

2a. In the **<u>past 3 months</u>**, how often have you used **tobacco** (For example: cigarettes, cigars or chewing tobacco)?

6 5 to 7 days a week
4 1 to 4 days a week
3 1 to 2 days a month
2 1 to 2 days in the past 3 months
0 Not used in the past 3 months
97 Don't know
98 Refuse to answer
99 Staff Only

[If yes to 1b]

2b. In the **<u>past 3 months</u>**, how often have you used **alcohol**?

6 5 to 7 days a week
4 1 to 4 days a week
3 1 to 2 days a month
2 1 to 2 days in the past 3 months
0 Not used in the past 3 months
97 Don't know
98 Refuse to answer
99 Staff Only

[If yes to 1c]

2c. In the **<u>past 3 months</u>**, how often have you used **marijuana**?

6 5 to 7 days a week
4 1 to 4 days a week
3 1 to 2 days a month
2 1 to 2 days in the past 3 months
0 Not used in the past 3 months
97 Don't know
98 Refuse to answer
99 Staff Only

[If yes 1d]

2d. In the **<u>past 3 months</u>**, how often have you used **cocaine** (For example: coke, crack)?

6 5 to 7 days a week
4 1 to 4 days a week
3 1 to 2 days a month
2 1 to 2 days in the past 3 months
0 Not used in the past 3 months
97 Don't know
98 Refuse to answer
99 Staff Only

[If yes to 1e]

2e. In the **<u>past 3 months</u>**, how often have you used **methamphetamines** (For example: *crank,* crystal meth, ecstasy or MDMA, *tweak*)?

6 5 to 7 days a week
4 1 to 4 days a week
3 1 to 2 days a month
2 1 to 2 days in the past 3 months
0 Not used in the past 3 months
97 Don't know
98 Refuse to answer
99 Staff Only

[If yes to 1f]

2f. In the **<u>past 3 months</u>**, how often have you used **inhalants** (For example: gasoline; glues; paint thinners; *poppers*, snappers or nitrous oxide, spray paint or other aerosol; *whippets*)?

6 5 to 7 days a week
4 1 to 4 days a week
3 1 to 2 days a month
2 1 to 2 days in the past 3 months
0 Not used in the past 3 months
97 Don't know
98 Refuse to answer
99 Staff Only

[If yes to 1g]

2g. In the **<u>past 3 months</u>**, how often have you used **hallucinogens** or "trip drugs" (For example: dextromethorphan, *DM* or *DXM*; coricidin C or *Robo*; herbal hallucinogens; ketamine, *K* or *special K*; LSD or *acid*; mushrooms; PCP or *angel dust*; peyote)?

6 5 to 7 days a week
4 1 to 4 days a week
3 1 to 2 days a month
2 1 to 2 days in the past 3 months
0 Not used in the past 3 months
97 Don't know
98 Refuse to answer
99 Staff Only

[If yes to 1h]

2h. In the **<u>past 3 months</u>**, how often have you used **heroin** or **opium**?

6 5 to 7 days a week
4 1 to 4 days a week
3 1 to 2 days a month
2 1 to 2 days in the past 3 months

0 Not used in the past 3 months
97 Don’t know
98 Refuse to answer
99 Staff Only

[If yes to 1i]

2i. In the **past 3 months**, how often have you used **GHB** or **GBH** (*G, Georgia Home Boy, Grievous Bodily Harm*)?

6 5 to 7 days a week
4 1 to 4 days a week
3 1 to 2 days a month
2 1 to 2 days in the past 3 months
0 Not used in the past 3 months
97 Don’t know
98 Refuse to answer
99 Staff Only

Information to respondent: ***We will now ask you if you have misused any type of prescription drug:***

- ***5 to 7 days a week***
- ***1 to 4 days a week***
- ***1 to 2 days a month***
- ***1 to 2 days in the past 3 months***
- ***Not misused in the past 3 months***

Please hit "next question"

[If yes to 1k]

2k. In the **past 3 months**, how often have you **misused** amphetamines (For example: Adderall; Concerta; Dexedrine; Focalin; Ritalin)?

6 5 to 7 days a week
4 1 to 4 days a week
3 1 to 2 days a month
2 1 to 2 days in the past 3 months
0 Not misused in the past 3 months
97 Don’t know
98 Refuse to answer
99 Staff Only

[If yes to 1l]

2l. In the **past 3 months**, how often have you **misused** benzodiazepines or "benzos" (For example: Ativan; Klonopin; Restoril; Valium; Xanax)?

6 5 to 7 days a week
4 1 to 4 days a week
3 1 to 2 days a month
2 1 to 2 days in the past 3 months

0 Not misused in the past 3 months
97 Don’t know
98 Refuse to answer
99 Staff Only

[If yes to 1m]

2m. In the **past 3 months**, how often have you **misused** barbiturates (For example: Butisol Sodium; Nembutal Sodium; Seconal Sodium; Solfoton)?

6 5 to 7 days a week
4 1 to 4 days a week
3 1 to 2 days a month
2 1 to 2 days in the past 3 months
0 Not misused in the past 3 months
97 Don’t know
98 Refuse to answer
99 Staff Only

[If yes to 1n]

2n. In the **past 3 months**, how often have you **misused** methadone or Suboxone (buprenorphine)?

6 5 to 7 days a week
4 1 to 4 days a week
3 1 to 2 days a month
2 1 to 2 days in the past 3 months
0 Not misused in the past 3 months
97 Don’t know
98 Refuse to answer
99 Staff Only

[If yes to 1o]

2o. In the **past 3 months**, how often have you **misused** prescription pain killers (For example: Darvocet; Demerol; Dilaudid; Fentora; Lortab; Oxycontin; Percocet; Vicodin)?

6 5 to 7 days a week
4 1 to 4 days a week
3 1 to 2 days a month
2 1 to 2 days in the past 3 months
0 Not misused in the past 3 months
97 Don’t know
98 Refuse to answer
99 Staff Only

Information to respondent: ***In the next few questions, we will ask if you have had a strong urge or craving for tobacco, alcohol or drugs in the past 3 months.***
Please hit “next question”

[If 2a>0]

3a. In the **past 3 months**, how often have you had a strong urge or craving to use **tobacco** (For example: cigarettes, cigars or chewing tobacco)?

6 5 to 7 days a week
5 1 to 4 days a week
4 1 to 2 days a month
3 1 to 2 days in the past 3 months
0 No strong urge or craving in the past 3 months
97 Don’t know
98 Refuse to answer
99 Staff Only

[If 2b>0]

3b. In the **past 3 months**, how often have you had a strong urge or craving to use **alcohol**?

6 5 to 7 days a week
5 1 to 4 days a week
4 1 to 2 days a month
3 1 to 2 days in the past 3 months
0 No strong urge or craving in the past 3 months
97 Don’t know
98 Refuse to answer
99 Staff Only

[If 2c>0]

3c. In the **past 3 months**, how often have you had a strong urge or craving to use **marijuana**?

6 5 to 7 days a week
5 1 to 4 days a week
4 1 to 2 days a month
3 1 to 2 days in the past 3 months
0 No strong urge or craving in the past 3 months
97 Don’t know
98 Refuse to answer
99 Staff Only

[If 2d>0]

3d. In the **past 3 months**, how often have you had a strong urge or craving to use **cocaine** (For example: coke, crack)?

6 5 to 7 days a week
5 1 to 4 days a week
4 1 to 2 days a month
3 1 to 2 days in the past 3 months
0 No strong urge or craving in the past 3 months
97 Don’t know
98 Refuse to answer
99 Staff Only

[If 2e>0]

3e. In the **<u>past 3 months</u>**, how often have you had a strong urge or craving to use **methamphetamines** (For example: *crank*, crystal meth, ecstasy or MDMA, *tweak*)?

6 5 to 7 days a week
5 1 to 4 days a week
4 1 to 2 days a month
3 1 to 2 days in the past 3 months
0 No strong urge or craving in the past 3 months
97 Don't know
98 Refuse to answer
99 Staff Only

[If 2f>0]

3f. In the **<u>past 3 months</u>**, how often have you had a strong urge or craving to use **inhalants** (For example: gasoline; glues; paint thinners; *poppers*, snappers or nitrous oxide, spray paint or other aerosol; *whippets*)?

6 5 to 7 days a week
5 1 to 4 days a week
4 1 to 2 days a month
3 1 to 2 days in the past 3 months
0 No strong urge or craving in the past 3 months
97 Don't know
98 Refuse to answer
99 Staff Only

[If 2g>0]

3g. In the **<u>past 3 months</u>**, how often have you had a strong urge or craving to use **hallucinogens** or "trip drugs" (For example: dextromethorphan, *DM* or *DXM*; coricidin C or *Robo*; herbal hallucinogens; ketamine, *K* or *special K*; LSD or *acid*; mushrooms; PCP or *angel dust*; peyote)?

6 5 to 7 days a week
5 1 to 4 days a week
4 1 to 2 days a month
3 1 to 2 days in the past 3 months
0 No strong urge or craving in the past 3 months
97 Don't know
98 Refuse to answer
99 Staff Only

[If 2h>0]

3h. In the **<u>past 3 months</u>**, how often have you had a strong urge or craving to use **heroin** or **opium**?

6 5 to 7 days a week
5 1 to 4 days a week
4 1 to 2 days a month

3 1 to 2 days in the past 3 months
0 No strong urge or craving in the past 3 months
97 Don’t know
98 Refuse to answer
99 Staff Only

[If 2i>0]
3i. In the **past 3 months**, how often have you had a strong urge or craving to use **GHB** or **GBH** (*G, Georgia Home Boy, Grievous Bodily Harm*)?
6 5 to 7 days a week
5 1 to 4 days a week
4 1 to 2 days a month
3 1 to 2 days in the past 3 months
0 No strong urge or craving in the past 3 months
97 Don’t know
98 Refuse to answer
99 Staff Only

[If 2k>0]
3k. In the **past 3 months**, how often have you had a strong urge or craving to use **amphetamines** (For example: Adderall; Concerta; Dexedrine; Focalin; Ritalin)?
6 5 to 7 days a week
5 1 to 4 days a week
4 1 to 2 days a month
3 1 to 2 days in the past 3 months
0 No strong urge or craving in the past 3 months
97 Don’t know
98 Refuse to answer
99 Staff Only

[If 2l>0]
3l. In the **past 3 months**, how often have you had a strong urge or craving to use **benzodiazepines** or "benzos" (For example: Ativan; Klonopin; Restoril; Valium; Xanax)?
6 5 to 7 days a week
5 1 to 4 days a week
4 1 to 2 days a month
3 1 to 2 days in the past 3 months
0 No strong urge or craving in the past 3 months
97 Don’t know
98 Refuse to answer
99 Staff Only

[If 2m>0]
3m. In the **past 3 months**, how often have you had a strong urge or craving to use **barbiturates** (For example: Butisol Sodium; Nembutal Sodium; Seconal Sodium; Solfoton)?

6 5 to 7 days a week
5 1 to 4 days a week
4 1 to 2 days a month
3 1 to 2 days in the past 3 months
0 No strong urge or craving in the past 3 months
97 Don't know
98 Refuse to answer
99 Staff Only

[If 2n>0]
3n. In the **past 3 months**, how often have you had a strong urge or craving to use **methadone** or **Suboxone** (buprenorphine)?

6 5 to 7 days a week
5 1 to 4 days a week
4 1 to 2 days a month
3 1 to 2 days in the past 3 months
0 No strong urge or craving in the past 3 months
97 Don't know
98 Refuse to answer
99 Staff Only

[If 2o>0]
3o. In the **past 3 months**, how often have you had a strong urge or craving to use prescription **pain killers** (For example: Darvocet; Demerol; Dilaudid; Fentora; Lortab; Oxycontin; Percocet; Vicodin)?

6 5 to 7 days a week
5 1 to 4 days a week
4 1 to 2 days a month
3 1 to 2 days in the past 3 months
0 No strong urge or craving in the past 3 months
97 Don't know
98 Refuse to answer
99 Staff Only

Information to respondent: ***Using tobacco, alcohol or drugs can cause people to have health, legal or financial problems or problems with friends, relatives, coworkers or other people.***

We will now ask you how often you have had these problems in the past 3 months. Please hit "next question"

[If 2a >0]

4a. In the **<u>past 3 months</u>**, how often has your use of **tobacco** (For example: cigarettes, cigars or chewing tobacco) caused health, legal or financial problems or problems with friends, relatives, coworkers or any other person?

7 5 to 7 days a week
6 1 to 4 days a week
5 1 to 2 days a month
4 1 to 2 days in the past 3 months
0 Not in the past 3 months
97 Don't know
98 Refuse to answer
99 Staff Only

[If 2b >0]

4b. In the **<u>past 3 months</u>**, how often has your use of **alcohol** caused health, legal or financial problems or problems with friends, relatives, coworkers or any other person?

7 5 to 7 days a week
6 1 to 4 days a week
5 1 to 2 days a month
4 1 to 2 days in the past 3 months
0 Not in the past 3 months
97 Don't know
98 Refuse to answer
99 Staff Only

[If 2c >0]

4c. In the **<u>past 3 months</u>**, how often has your use of **marijuana** caused health, legal or financial problems or problems with friends, relatives, coworkers or any other person?

7 5 to 7 days a week
6 1 to 4 days a week
5 1 to 2 days a month
4 1 to 2 days in the past 3 months
0 Not in the past 3 months
97 Don't know
98 Refuse to answer
99 Staff Only

[If 2d >0]

4d. In the **<u>past 3 months</u>**, how often has your use of **cocaine** (For example: coke, crack) caused health, legal, financial problems or problems with friends, relatives, coworkers or other people?

7 5 to 7 days a week
6 1 to 4 days a week
5 1 to 2 days a month
4 1 to 2 days in the past 3 months
0 Not in the past 3 months

97 Don’t know
98 Refuse to answer
99 Staff Only

[If 2e >0]

4e. In the **<u>past 3 months</u>**, how often has your use of **methamphetamines** (For example: *crank*, crystal meth, ecstasy or MDMA, *tweak*) caused health, legal or financial problems or problems with friends, relatives, coworkers or any other person?

7 5 to 7 days a week
6 1 to 4 days a week
5 1 to 2 days a month
4 1 to 2 days in the past 3 months
0 Not in the past 3 months
97 Don’t know
98 Refuse to answer
99 Staff Only

[If 2f >0]

4f. In the **<u>past 3 months</u>**, how often has your use of **inhalants** (For example: gasoline; glues; paint thinners; *poppers*, snappers or nitrous oxide, spray paint or other aerosol; *whippets*) caused health, legal or financial problems or problems with friends, relatives, coworkers or any other person?

7 5 to 7 days a week
6 1 to 4 days a week
5 1 to 2 days a month
4 1 to 2 days in the past 3 months
0 Not in the past 3 months
97 Don’t know
98 Refuse to answer
99 Staff Only

[If 2g >0]

4g. In the **<u>past 3 months</u>**, how often has your use of **hallucinogens** or “trip drugs” (For example: dextromethorphan, *DM* or *DXM*; coricidin C or *Robo*; herbal hallucinogens; ketamine, *K* or *special K*; LSD or *acid*; mushrooms; PCP or *angel dust*; peyote) caused health, legal or financial problems or problems with friends, relatives, coworkers or any other person?

7 5 to 7 days a week
6 1 to 4 days a week
5 1 to 2 days a month
4 1 to 2 days in the past 3 months
0 Not in the past 3 months
97 Don’t know
98 Refuse to answer
99 Staff Only

[If 2h >0]

4h. In the **past 3 months**, how often has your use of **heroin** or **opium** caused health, legal or financial problems or problems with friends, relatives, coworkers or any other person?

7 5 to 7 days a week
6 1 to 4 days a week
5 1 to 2 days a month
4 1 to 2 days in the past 3 months
0 Not in the past 3 months
97 Don't know
98 Refuse to answer
99 Staff Only

[If 2i >0]

4i. In the **past 3 months**, how often has your use of **GHB** or **GBH** (G, Georgia Home Boy, Grievous Bodily Harm) caused health, legal or financial problems or problems with friends, relatives, coworkers or any other person?

7 5 to 7 days a week
6 1 to 4 days a week
5 1 to 2 days a month
4 1 to 2 days in the past 3 months
0 Not in the past 3 months
97 Don't know
98 Refuse to answer
99 Staff Only

[If 2k >0]

4k. In the **past 3 months**, how often has your use of **amphetamines** (For example: Adderall; Concerta; Dexedrine; Focaline; Ritalin) caused health, legal or financial problems or problems with friends, relatives, coworkers or any other person?

7 5 to 7 days a week
6 1 to 4 days a week
5 1 to 2 days a month
4 1 to 2 days in the past 3 months
0 Not in the past 3 months
97 Don't know
98 Refuse to answer
99 Staff Only

[If 2l >0]

4l. In the **past 3 months**, how often has your use of **benzodiazepines** or "benzos" (For example: Ativan; Klonopin; Restoril; Valium; Xanax) caused health, legal or financial problems or problems with friends, relatives, coworkers or any other person?

7 5 to 7 days a week
6 1 to 4 days a week
5 1 to 2 days a month

4 1 to 2 days in the past 3 months
0 Not in the past 3 months
97 Don’t know
98 Refuse to answer
99 Staff Only

[If 2m >0]

4m. In the **<u>past 3 months</u>**, how often has your use of **barbiturates** (For example: Butisol Sodium; Nembutal Sodium; Seconal Sodium; Solfoton) caused health, legal or financial problems or problems with friends, relatives, coworkers or any other person?

7 5 to 7 days a week
6 1 to 4 days a week
5 1 to 2 days a month
4 1 to 2 days in the past 3 months
0 Not in the past 3 months
97 Don’t know
98 Refuse to answer
99 Staff Only

[If 2n >0]

4n. In the **<u>past 3 months</u>**, how often has your use of **methadone** or **Suboxone** (buprenorphine) caused health, legal or financial problems or problems with friends, relatives, coworkers or any other person?

7 5 to 7 days a week
6 1 to 4 days a week
5 1 to 2 days a month
4 1 to 2 days in the past 3 months
0 Not in the past 3 months
97 Don’t know
98 Refuse to answer
99 Research Assistant only

[If 2o >0]

4o. In the **<u>past 3 months</u>**, how often has your use of prescription **pain killers** (For example: Darvocet; Demerol; Dilaudid; Fentora; Lortab; Oxycontin; Percocet; Vicodin) caused health, legal or financial problems or problems with friends, relatives, coworkers or any other person?

7 5 to 7 days a week
6 1 to 4 days a week
5 1 to 2 days a month
4 1 to 2 days in the past 3 months
0 Not in the past 3 months
97 Don’t know
98 Refuse to answer
99 Staff Only

Information to respondent: ***Using tobacco, alcohol or drugs can cause people to fail to do the things that are usually expected of them.***

We will now ask you how often your use of alcohol, tobacco or drugs has caused you to fail to do the things that are usually expected of you in the past 3 months.

Please hit "next question"

[If 2a >0]

5a. In the **past 3 months**, how often have you failed to do the things that are usually expected of you because of your use of **tobacco** (For example: cigarettes, cigars or chewing tobacco)?

8 5 to 7 days a week
7 1 to 4 days a week
6 1 to 2 days a month
5 1 to 2 days in the past 3 months
0 Not in the past 3 months
97 Don't know
98 Refuse to answer
99 Staff Only

[If 2b >0]

5b. In the **past 3 months**, how often have you failed to do the things that are usually expected of you because of your use of **alcohol**?

8 5 to 7 days a week
7 1 to 4 days a week
6 1 to 2 days a month
5 1 to 2 days in the past 3 months
0 Not in the past 3 months
97 Don't know
98 Refuse to answer
99 Staff Only

[If 2c >0]

5c. In the **past 3 months**, how often have you failed to do the things that are usually expected of you because of your use of **marijuana**?

8 5 to 7 days a week
7 1 to 4 days a week
6 1 to 2 days in past month
5 1 to 2 days in the past 3 months
0 Not in the past 3 months
97 Don't know
98 Refuse to answer
99 Staff Only

[If 2d >0]

5d. In the **past 3 months**, how often have you failed to do the things that are usually expected of you because of your use of **cocaine** (For example: coke, crack)?

8 5 to 7 days a week
7 1 to 4 days a week
6 1 to 2 days in past month
5 1 to 2 days in the past 3 months
0 Not in the past 3 months
97 Don't know
98 Refuse to answer
99 Staff Only

[If 2e >0]

5e. In the **past 3 months**, how often have you failed to do the things that are usually expected of you because of your use of **methamphetamines** (For example: *crank*, crystal meth, ecstasy or MDMA, *tweak*)?

8 5 to 7 days a week
7 1 to 4 days a week
6 1 to 2 days in past month
5 1 to 2 days in the past 3 months
0 Not in the past 3 months
97 Don't know
98 Refuse to answer
99 Staff Only

[If 2f >0]

5f. In the **past 3 months**, how often have you failed to do the things that are usually expected of you because of your use of **inhalants** (For example: gasoline; glues; paint thinners; *poppers*, snappers or nitrous oxide, spray paint or other aerosol; *whippets*)?

8 5 to 7 days a week
7 1 to 4 days a week
6 1 to 2 days in past month
5 1 to 2 days in the past 3 months
0 Not in the past 3 months
97 Don't know
98 Refuse to answer
99 Staff Only

[If 2g >0]

5g. In the **past 3 months**, how often have you failed to do the things that are usually expected of you because of your use of **hallucinogens** or "trip drugs" (For example: dextromethorphan, *DM* or *DXM*; coricidin C or *Robo*; herbal hallucinogens; ketamine, *K* or *special K*; LSD or *acid*; mushrooms; PCP or *angel dust*; peyote)?

8 5 to 7 days a week
7 1 to 4 days a week
6 1 to 2 days in past month

5 1 to 2 days in the past 3 months
0 Not in the past 3 months
97 Don't know
98 Refuse to answer
99 Staff Only

[If 2h >0]

5h. In the **past 3 months**, how often have you failed to do the things that are usually expected of you because of your use of **heroin** or **opium**?

8 5 to 7 days a week
7 1 to 4 days a week
6 1 to 2 days in past month
5 1 to 2 days in the past 3 months
0 Not in the past 3 months
97 Don't know
98 Refuse to answer
99 Staff Only

[If 2i >0]

5i. In the **past 3 months**, how often have you failed to do the things that are usually expected of you because of your use of **GHB** or **GBH** (*G, Georgia Home Boy, Grievous Bodily Harm*)?

8 5 to 7 days a week
7 1 to 4 days a week
6 1 to 2 days in past month
5 1 to 2 days in the past 3 months
0 Not in the past 3 months
97 Don't know
98 Refuse to answer
99 Staff Only

[If 2k >0]

5k. In the **past 3 months**, how often have you failed to do the things that are usually expected of you because of your use of **amphetamines** (For example: Adderall; Concerta; Dexedrine; Focalin; Ritalin)?

8 5 to 7 days a week
7 1 to 4 days a week
6 1 to 2 days in past month
5 1 to 2 days in the past 3 months
0 Not in the past 3 months
97 Don't know
98 Refuse to answer
99 Staff Only

[If 2l >0]

5l. In the **past 3 months**, how often have you failed to do the things that are usually expected of you because of your use of **benzodiazepines** or "benzos" (For example: Ativan; Klonopin ; Restoril; Valium; Xanax)?

8 5 to 7 days a week
7 1 to 4 days a week
6 1 to 2 days in past month
5 1 to 2 days in the past 3 months
0 Not in the past 3 months
97 Don't know
98 Refuse to answer
99 Staff Only

[If 2m >0]

5m. In the **past 3 months**, how often have you failed to do the things that are usually expected of you because of your use of **barbiturates** (For example: Butisol Sodium; Nembutal Sodium; Seconal Sodium; Solfoton)?

8 5 to 7 days a week
7 1 to 4 days a week
6 1 to 2 days in past month
5 1 to 2 days in the past 3 months
0 Not in the past 3 months
97 Don't know
98 Refuse to answer
99 Staff Only

[If 2n >0]

5n. In the **past 3 months**, how often have you failed to do the things that are usually expected of you because of your use of **methadone** or **Suboxone** (buprenorphine)?

8 5 to 7 days a week
7 1 to 4 days a week
6 1 to 2 days in past month
5 1 to 2 days in the past 3 months
0 Not in the past 3 months
97 Don't know
98 Refuse to answer
99 Staff Only

[If 2o >0]

5o. In the **past 3 months**, how often have you failed to do the things that are usually expected of you because of your use of prescription **pain killers** (For example: Darvocet; Demerol; Dilaudid; Fentora; Lortab; Oxycontin; Percocet; Vicodin)?

8 5 to 7 days a week
7 1 to 4 days a week
6 1 to 2 days in past month
5 1 to 2 days in the past 3 months
0 Not in the past 3 months

97 Don’t know
98 Refuse to answer
99 Staff Only

<u>Information to respondent:</u> ***In the next few questions, we will ask you if a friend, relative or anyone else has ever said they were concerned about your use of tobacco, alcohol or drugs.***

Please hit "next question"

[If 1a is yes]

6a. Has a friend, relative or anyone else **<u>ever</u>** said they were concerned about your use of **tobacco** (For example: cigarettes, cigars or chewing tobacco)?

3 Yes
0 No
97 Don’t know
98 Refuse to answer
99 Staff Only

[If 6a=YES and 2a>0]

6a1. In the **<u>past 3 months</u>**, has a friend, relative or anyone else said they were concerned about your use of **tobacco** (For example: cigarettes, cigars or chewing tobacco)?

3 Yes
0 No
97 Don’t know
98 Refuse to answer
99 Staff Only

[If 1b is yes]

6b. Has a friend, relative or anyone else **ever** said they were concerned about your use of **alcohol**?

3 Yes
0 No
97 Don’t know
98 Refuse to answer
99 Staff Only

[If 6b=YES and 2b>0]

6b1. In the **<u>past 3 months</u>**, has a friend, relative or anyone else said they were concerned about your use of **alcohol**?

3 Yes
0 No
97 Don’t know
98 Refuse to answer
99 Staff Only

[If 1c is yes]

6c. Has a friend, relative or anyone else **ever** said they were concerned about your use of **marijuana**?
3 Yes
0 No
97 Don't know
98 Refuse to answer
99 Staff Only

[If 6c=YES and 2c>0]
6c1. In the **<u>past 3 months</u>**, has a friend, relative or anyone else said they were concerned about your use of **marijuana**?
3 Yes
0 No
97 Don't know
98 Refuse to answer
99 Staff Only

[If 1d is yes]
6d. Has a friend, relative or anyone else **ever** said they were concerned about your use of **cocaine** (For example: coke, crack)?
3 Yes
0 No
97 Don't know
98 Refuse to answer
99 Staff Only

[If 6d=YES and 2d>0]
6d1. In the **<u>past 3 months</u>**, has a friend, relative or anyone else said they were concerned about your use of **cocaine** (For example: coke, crack)?
3 Yes
0 No
97 Don't know
98 Refuse to answer
99 Staff Only

[If 1e is yes]
6e. Has a relative or anyone else **ever** said they were concerned about your use of **methamphetamines** (For example: *crank*, crystal meth, ecstasy or MDMA, *tweak*)?
3 Yes
0 No
97 Don't know
98 Refuse to answer
99 Staff Only

[If 6e=YES and 2e>0]

6e1. In the **<u>past 3 months</u>**, has a friend, relative or anyone else said they were concerned about your use of **methamphetamines** (For example: *crank*, crystal meth, ecstasy or MDMA, *tweak*)?

3 Yes
0 No
97 Don't know
98 Refuse to answer
99 Staff Only

[If 1f is yes]

6f. Has a friend, relative or anyone else **ever** said they were concerned about your use of **inhalants** (For example: gasoline; glues; paint thinners; *poppers*, snappers or nitrous oxide, spray paint or other aerosol; *whippets*)?

3 Yes
0 No
97 Don't know
98 Refuse to answer
99 Staff Only

[If 6f=YES and 2f>0]

6f1. In the **<u>past 3 months</u>**, has a friend, relative or anyone else said they were concerned about your use of **inhalants** (For example: gasoline; glues; paint thinners; *poppers*, snappers or nitrous oxide, spray paint or other aerosol; *whippets*)?

3 Yes
0 No
97 Don't know
98 Refuse to answer
99 Staff Only

[If 1g is yes]

6g. Has a friend, relative or anyone else **ever** said they were concerned about your use of **hallucinogens** or "trip drugs" (For example: dextromethorphan, *DM* or *DXM*; coricidin C or *Robo*; herbal hallucinogens; ketamine, *K* or *special K*; LSD or *acid*; mushrooms; PCP or *angel dust*; peyote)?

3 Yes
0 No
97 Don't know
98 Refuse to answer
99 Staff Only

[If 6g=YES and 2g>0]

6g1. In the **<u>past 3 months</u>**, has a friend, relative or anyone else said they were concerned about your use of **hallucinogens** or "trip drugs" (For example: dextromethorphan, *DM* or *DXM*; coricidin C or *Robo*; herbal hallucinogens; ketamine, *K* or *special K*; LSD or *acid*; mushrooms; PCP or *angel dust*; peyote)

3 Yes
0 No

97 Don’t know
98 Refuse to answer
99 Staff Only

[If 1h is yes]

6h. Has a friend, relative or anyone else **ever** said they were concerned about your use of **heroin** or **opium**?

3 Yes
0 No
97 Don’t know
98 Refuse to answer
99 Staff Only

[If 6h=YES and 2h>0]

6h1. In the **past 3 months**, has a friend, relative or anyone else said they were concerned about your use of **heroin** or **opium**?

3 Yes
0 No
97 Don’t know
98 Refuse to answer
99 Staff Only

[If 1i is yes]

6i. Has a friend, relative or anyone else **ever** said they were concerned about your use of **GHB** or **GBH** (*G, Georgia Home Boy, Grievous Bodily Harm*)?

3 Yes
0 No
97 Don’t know
98 Refuse to answer
99 Staff Only

[If 6i=YES and 2i>0]

6i1. In the **past 3 months**, has a friend, relative or anyone else said they were concerned about your use of **GHB** or **GBH** (*G, Georgia Home Boy, Grievous Bodily Harm*)?

3 Yes
0 No
97 Don’t know
98 Refuse to answer
99 Staff Only

[If 1j is yes]

6j. Has a friend, relative or anyone else **ever** said they were concerned about your use of **amphetamines** (For example: Adderall; Concerta; Dexedrine; Focalin; Ritalin)?

3 Yes
0 No
97 Don’t know
98 Refuse to answer
99 Staff Only

[If 6j=YES and 2j>0]

6j1. In the **past 3 months**, has a friend, relative or anyone else said they were concerned about your use of **amphetamines** (For example: Adderall; Concerta; Dexedrine; Focalin; Ritalin)?

3 Yes
0 No
97 Don’t know
98 Refuse to answer
99 Staff Only

[If 1k is yes]

6k. Has a friend, relative or anyone else **ever** said they were concerned about your use of **benzodiazepines** or "benzos" (For example: Ativan; Klonopin; Restoril; Valium; Xanax)?

3 Yes
0 No
97 Don’t know
98 Refuse to answer
99 Staff Only

[If 6k=YES and 2k>0]

6k1. In the **past 3 months**, has a friend, relative or anyone else said they were concerned about your use of **benzodiazepines** or "benzos" (For example: Ativan; Klonopin; Restoril; Valium; Xanax)?

3 Yes
0 No
97 Don’t know
98 Refuse to answer
99 Staff Only

[If 1l is yes]

6l. Has a friend, relative or anyone else **ever** said they were concerned about your use of **barbiturates** (For example: Butisol Sodium; Nembutal Sodium; Seconal Sodium; Solfoton)?

3 Yes
0 No
97 Don’t know
98 Refuse to answer
99 Staff Only

[If 6l=YES and 2l>0]

6l1. In the **past 3 months**, has a friend, relative or anyone else said they were concerned about your use of **barbiturates** (For example: Butisol Sodium; Nembutal Sodium; Seconal Sodium; Solfoton)?
3 Yes
0 No
97 Don't know
98 Refuse to answer
99 Staff Only

[If 1m is yes]
6m. Has a friend, relative or anyone else **ever** said they were concerned about your use of **methadone** or **Suboxone** (buprenorphine)?
3 Yes
0 No
97 Don't know
98 Refuse to answer
99 Staff Only

[If 6m=YES and 2m>0]
6m1. In the **past 3 months**, has a friend, relative or anyone else said they were concerned about your use of **methadone** or **Suboxone** (buprenorphine)?
3 Yes
0 No
97 Don't know
98 Refuse to answer
99 Staff Only

[If 1n is yes]
6n. Has a friend, relative or anyone else **ever** said they were concerned about your use of prescription **pain killers** (For example: Darvocet; Demerol; Dilaudid; Fentora; Lortab; Oxycontin ; Percocet; Vicodin)?
3 Yes
0 No
97 Don't know
98 Refuse to answer
99 Staff Only

[If 6n=YES and 2n>0]
6n1. In the **past 3 months**, has a friend, relative or anyone else said they were concerned about your use of prescription **pain killers** (For example: Darvocet; Demerol; Dilaudid; Fentora; Lortab; Oxycontin ; Percocet; Vicodin)?
3 Yes
0 No
97 Don't know
98 Refuse to answer
99 Staff Only

<u>Information to respondent:</u> ***In the next few questions, we will ask you if you have ever tried to control, cut down or stop using tobacco, alcohol or drugs but could not.***

Please hit "next question"

[If 1a is yes]

7a. Have you ever tried to control, cut down or stop using **tobacco** (For example: cigarettes, cigars or chewing tobacco) but could not?

- 3 Yes
- 0 No
- 97 Don't know
- 98 Refuse to answer
- 99 Staff Only

[If 7a=YES and 2a>0]

7a1. In the **<u>past 3 months</u>**, have you tried to control, cut down or stop using **tobacco** (For example: cigarettes, cigars or chewing tobacco) but could not?

- 3 Yes
- 0 No
- 97 Don't know
- 98 Refuse to answer
- 99 Staff Only

[If 1b is yes]

7b. Have you ever tried to control, cut down or stop using **alcohol** but could not?

- 3 Yes
- 0 No
- 97 Don't know
- 98 Refuse to answer
- 99 Staff Only

[If 7b=YES and 2b>0]

7b1. In the **<u>past 3 months</u>**, have you tried to control, cut down or stop using **alcohol** but could not?

- 3 Yes
- 0 No
- 97 Don't know
- 98 Refuse to answer
- 99 Staff Only

[If 1c is yes]

7c. Have you ever tried to control, cut down or stop using **marijuana** but could not?

- 3 Yes
- 0 No
- 97 Don't know
- 98 Refuse to answer
- 99 Staff Only

[If 7c=YES and 2c>0]

7c1. In the **past 3 months**, have you tried to control, cut down or stop using **marijuana** but could not?

3 Yes
0 No
97 Don't know
98 Refuse to answer
99 Staff Only

[If 1d is yes]

7d. Have you ever tried to control, cut down or stop using **cocaine** (For example: coke, crack) but could not?

3 Yes
0 No
97 Don't know
98 Refuse to answer
99 Staff Only

[If 7d=YES and 2d>0]

7d1. In the **past 3 months**, have you tried to control, cut down or stop using **cocaine** (For example: coke, crack) but could not?

3 Yes
0 No
97 Don't know
98 Refuse to answer
99 Staff Only

[If 1e is yes]

7e. Have you ever tried to control, cut down or stop using **methamphetamines** (For example: *crank*, crystal meth, ecstasy or MDMA, *tweak*) but could not?

3 Yes
0 No
97 Don't know
98 Refuse to answer
99 Staff Only

[If 7e=YES and 2e>0]

7e1. In the **past 3 months**, have you tried to control, cut down or stop using **methamphetamines** (For example: *crank*, crystal meth, ecstasy or MDMA, *tweak*) but could not?

3 Yes
0 No
97 Don't know
98 Refuse to answer
99 Staff Only

[If 1f is yes]

7f. Have you tried to control, cut down or stop using **inhalants** (For example: gasoline; glues; paint thinners; *poppers,* snappers or nitrous oxide, spray paint or other aerosol; *whippets*) but could not?

3 Yes
0 No
97 Don’t know
98 Refuse to answer
99 Staff Only

[If 7f=YES and 2f>0]

7f1. In the **<u>past 3 months</u>**, have you tried to control, cut down or stop using **inhalants** (For example: gasoline; glues; paint thinners; *poppers*, snappers or nitrous oxide, spray paint or other aerosol; *whippets*) but could not?

3 Yes
0 No
97 Don’t know
98 Refuse to answer
99 Staff Only

[If 1g is yes]

7g. Have you ever tried to control, cut down or stop using **hallucinogens** or “trip drugs” (For example: dextromethorphan, *DM* or *DXM*; coricidin C or *Robo*; herbal hallucinogens; ketamine, *K* or *special K*; LSD or *acid*; mushrooms; PCP or *angel dust*; peyote) but could not?

3 Yes
0 No
97 Don’t know
98 Refuse to answer
99 Staff Only

[If 7g=YES and 2g>0]

7g1. In the **<u>past 3 months</u>**, have you tried to control, cut down or stop using **hallucinogens** or “trip drugs” (For example: dextromethorphan, *DM* or *DXM*; coricidin C or *Robo*; herbal hallucinogens; ketamine, *K* or *special K*; LSD or *acid*; mushrooms; PCP or *angel dust*; peyote) but could not?

3 Yes
0 No
97 Don’t know
98 Refuse to answer
99 Staff Only

[If 1h is yes]

7h. Have you ever tried to control, cut down or stop using **heroin** or **opium** but could not?

3 Yes
0 No
97 Don’t know

98 Refuse to answer
99 Staff Only

[If 7h=YES and 2h>0]

7h1. In the **past 3 months**, have you tried to control, cut down or stop using **heroin** or **opium** but could not?

3 Yes
0 No
97 Don't know
98 Refuse to answer
99 Staff Only

[If 1i is yes]

7i. Have you ever tried to control, cut down or stop using **GHB** or **GBH** (G, Georgia Home Boy, Grievous Bodily Harm) but could not?

3 Yes
0 No
97 Don't know
98 Refuse to answer
99 Staff Only

[If 7i=YES and 2i>0]

7i1. In the **past 3 months**, have you tried to control, cut down or stop using **GHB** or **GBH** (G, Georgia Home Boy, Grievous Bodily Harm) but could not?

3 Yes
0 No
97 Don't know
98 Refuse to answer
99 Staff Only

[If 1k is yes]

7k. Have you ever tried to control, cut down or stop using **amphetamines** (For example: Adderall; Concerta; Dexedrine; Focalin; Ritalin) but could not?

3 Yes
0 No
97 Don't know
98 Refuse to answer
99 Staff Only

[If 7k=YES and 2k>0]

7k1. In the **past 3 months**, have you tried to control, cut down or stop using **amphetamines** (For example: Adderall; Concerta; Dexedrine; Focalin; Ritalin) but could not?

3 Yes
0 No
97 Don't know
98 Refuse to answer

99 Staff Only

[If 1l is yes]

7l. Have you ever tried to control, cut down or stop using **benzodiazepines** or "benzos" (For example: Ativan; Klonopin; Restoril; Valium; Xanax) but could not?

3 Yes
0 No
97 Don't know
98 Refuse to answer
99 Staff Only

[If 7l=YES and 2l>0]

7l1. In the **past 3 months**, have you tried to control, cut down or stop using **benzodiazepines** or "benzos" (For example: Ativan; Klonopin; Restoril; Valium; Xanax) but could not?

3 Yes
0 No
97 Don't know
98 Refuse to answer
99 Staff Only

[If 1m is yes]

7m. Have you ever tried to control, cut down or stop using **barbiturates** (For example: Butisol Sodium; Nembutal Sodium; Seconal Sodium; Solfoton) but could not?

3 Yes
0 No
97 Don't know
98 Refuse to answer
99 Staff Only

[If 7m=YES and 2m>0]

7m1. In the **past 3 months**, have you tried to control, cut down or stop using **barbiturates** (For example: Butisol Sodium; Nembutal Sodium; Seconal Sodium; Solfoton) but could not?

3 Yes
0 No
97 Don't know
98 Refuse to answer
99 Staff Only

[If 1n is yes]

7n. Have you ever tried to control, cut down or stop using **methadone** or **Suboxone** (buprenorphine) but could not?

3 Yes
0 No
97 Don't know

98 Refuse to answer
99 Staff Only

[If 7n=YES and 2n>0]

7n1. In the **past 3 months**, have you tried to control, cut down or stop using **methadone** or **Suboxone** (buprenorphine) but could not?

3 Yes
0 No
97 Don't know
98 Refuse to answer
99 Staff Only

[If 1o is yes]

7o. Have you ever tried to control, cut down or stop using prescription **pain killers** (For example: Darvocet; Demerol; Dilaudid; Fentora; Lortab; Oxycontin; Percocet; Vicodin) but could not?

3 Yes
0 No
97 Don't know
98 Refuse to answer
99 Staff Only

[If 7o=YES and 2o>0]

7o1. In the **past 3 months**, have you tried to control, cut down or stop using prescription **pain killers** (For example: Darvocet; Demerol; Dilaudid; Fentora; Lortab; Oxycontin; Percocet; Vicodin) but could not?

3 Yes
0 No
97 Don't know
98 Refuse to answer
99 Staff Only

8. Have you ever used any drug by injection for **non-medical use**?

By "**non-medical use**" we mean injecting drugs to get high or to help take away the bad effects of other drugs. Injection drugs may be street drugs such as heroin, cocaine or crystal meth. Or, injection drugs could have been prescribed but were not meant to be injected, such as Percocet.

1 Yes
0 No
97 Don't know
98 Refuse to answer
99 Staff Only

[If 8 is yes]

8a. In the **past 3 months**, have you used any drug by injection for **non-medical use**?

By “**non-medical use**” we mean injecting drugs to get high or to help take away the bad effects of other drugs. Injection drugs may be street drugs such as heroin, cocaine or crystal meth. Or, injection drugs could have been prescribed but were not meant to be injected, such as Percocet.

1 Yes
0 No
97 Don’t know
98 Refuse to answer
99 Staff Only

Information to respondent: ***We will now ask you about the drugs you have used in the past 3 months.***

Please hit “next question”

[If 2d, 2c, 2f, 2g and 2h are >0]

9. Which of the following drugs have you used in the **past 3 months**?
Please check all the ones you have used and hit “next question” to continue.
2__ cocaine
3__ crack
4__ crystal meth
5__ ecstasy or MDMA
6__ gasoline
7__ glues
8__ paint thinners
9__ poppers/snappers (nitrious oxide)
10__ spray paint or other aerosols
11__ whippets (nitrous oxide)
12__ LSD
13__mushrooms
14__dextromethorphan (DM, DXM)
15___coricidin C (Robo)
16___herbal halucinogens
17__ ketamine (K, special K)
18__ PCP
19__ peyote
20__ heroin
21__ opium
97 Don’t know
98 Refuse to answer
99 Staff Only

[If 2c>0]

10. Was **any** of the **marijuana** you used in the **past 3 months** prescribed to **YOU**?

1 No, none of it was prescribed to ME
2 Yes, some of it was prescribed to ME
3 Yes, all of it was prescribed to ME
97 Don’t know
98 Refuse to answer
99 Staff Only

[If 2k>0]
11. Which of the following **amphetamines** have you **misused** in the **past 3 months**? Please check all the ones you have **misused** and hit “next question” to continue**.**

1__ Adderall (amphetamine salts)
2__ Concerta (methylphenidate)
3__ Dexedrine (dextroamphetamine)
4__ Focalin (dexmethylphenidate)
5__ Methylin, Metadate or Ritalin (methylphenidate)
97 Don’t know
98 Refuse to answer
99 Staff Only

[If 11=1]
11a. Was **any** of the **Adderall** (amphetamine salts) you **misused** in the **past 3 months** prescribed to **YOU**?
1 No, none of it was prescribed to ME
2 Yes, some of it was prescribed to ME
3 Yes, all of it was prescribed to ME
97 Don’t know
98 Refuse to answer
99 Staff Only

[If 11=2]
11b. Was **any** of the **Concerta** (methylphenidate) you **misused** in the **past 3 months** prescribed to **YOU**?
1 No, none of it was prescribed to ME
2 Yes, some of it was prescribed to ME
3 Yes, all of it was prescribed to ME
97 Don’t know
98 Refuse to answer
99 Staff Only

[If 11=3]
11c. Was **any** of the **Dexedrine** (dextroamphetamine) you **misused** in the **past 3 months** prescribed to **YOU**?
1 No, none of it was prescribed to ME
2 Yes, some of it was prescribed to ME
3 Yes, all of it was prescribed to ME
97 Don’t know

98 Refuse to answer
99 Staff Only

[If 11=4]
11d. Was **<u>any</u>** of the **Focalin** (dexmethylphenidate) you **misused** in the **<u>past 3 months</u>** prescribed to **<u>YOU</u>**?
1 No, none of it was prescribed to ME
2 Yes, some of it was prescribed to ME
3 Yes, all of it was prescribed to ME
97 Don't know
98 Refuse to answer
99 Staff Only

[If 11=5]
11e. Was **<u>any</u>** of the **Methylin, Metadate or Ritalin** (methylphenidate) you **misused** in the **<u>past 3 months</u>** prescribed to **<u>YOU</u>**?
1 No, none of it was prescribed to ME
2 Yes, some of it was prescribed to ME
3 Yes, all of it was prescribed to ME
97 Don't know
98 Refuse to answer
99 Staff Only

[If 2l>0]
12. Which of the following **benzodiazepines** have **misused** in the **<u>past 3 months</u>**? Please check all the ones you have **misused** and hit "next question" to continue.
1__ Ativan (lorazepam)
2__ Doral (quazepam)
3__ Dalmane (flurazepam)
4__ Halcion (triazolam)
5__ Klonopin (clonazepam)
6__ Librium (chlordiazepoxide)
7__ Restoril (temazepam)
8__ Tranxene (clorazepate)
9__ Valium (diazepam)
10__ Xanax (alprazolam)
97 Don't know
98 Refuse to answer
99 Staff Only

[If 12=1]
12a. Was **<u>any</u>** of the **Ativan** (lorazepam) you **misused** in the **<u>past 3 months</u>** prescribed to **<u>YOU</u>**?
1 No, none of it was prescribed to ME
2 Yes, some of it was prescribed to ME

3 Yes, all of it was prescribed to ME
97 Don't know
98 Refuse to answer
99 Staff Only

[If 12=2]
12b. Was **<u>any</u>** of the **Doral** (quazepam) you **misused** in the **<u>past 3 months</u>** prescribed to **<u>YOU</u>**?
1 No, none of it was prescribed to ME
2 Yes, some of it was prescribed to ME
3 Yes, all of it was prescribed to ME
97 Don't know
98 Refuse to answer
99 Staff Only

[If 12=3]
12c. Was **<u>any</u>** of the **Dalmane** (flurazepam) you **misused** in the **<u>past 3 months</u>** prescribed to **<u>YOU</u>**?
1 No, none of it was prescribed to ME
2 Yes, some of it was prescribed to ME
3 Yes, all of it was prescribed to ME
97 Don't know
98 Refuse to answer
99 Staff Only

[If 12=4]
12d. Was **<u>any</u>** of the **Halcion** (triazolam) you **misused** in the **<u>past 3 months</u>** prescribed to **<u>YOU</u>**?
1 No, none of it was prescribed to ME
2 Yes, some of it was prescribed to ME
3 Yes, all of it was prescribed to ME
97 Don't know
98 Refuse to answer
99 Staff Only

[If 12=5]
12e. Was **<u>any</u>** of the **Klonopin** (clonazepam) you **misused** in the **<u>past 3 months</u>** prescribed to **<u>YOU</u>**?
1 No, none of it was prescribed to ME
2 Yes, some of it was prescribed to ME
3 Yes, all of it was prescribed to ME
97 Don't know
98 Refuse to answer
99 Staff Only

[If 12=6]

12f. Was **<u>any</u>** of the **Librium** (chlordiazepoxide) you **misused** in the **<u>past 3 months</u>** prescribed to **<u>YOU</u>**?

1 No, none of it was prescribed to ME
2 Yes, some of it was prescribed to ME
3 Yes, all of it was prescribed to ME
97 Don't know
98 Refuse to answer
99 Staff Only

[If 12=7]
12g. Was **<u>any</u>** of the **Restoril** (temazepam) you **misused** in the **<u>past 3 months</u>** prescribed to **<u>YOU</u>**?

1 No, none of it was prescribed to ME
2 Yes, some of it was prescribed to ME
3 Yes, all of it was prescribed to ME
97 Don't know
98 Refuse to answer
99 Staff Only

[If 12=8]
12h. Was **<u>any</u>** of the **Tranxene** (clorazepate) you **misused** in the **<u>past 3 months</u>** prescribed to **<u>YOU</u>**?

1 No, none of it was prescribed to ME
2 Yes, some of it was prescribed to ME
3 Yes, all of it was prescribed to ME
97 Don't know
98 Refuse to answer
99 Staff Only

[If 12=9]
12i. Was **<u>any</u>** of the **Valium** (diazepam) you **misused** in the **<u>past 3 months</u>** prescribed to **<u>YOU</u>**?

1 No, none of it was prescribed to ME
2 Yes, some of it was prescribed to ME
3 Yes, all of it was prescribed to ME
97 Don't know
98 Refuse to answer
99 Staff Only

[If 12=10]
12j. Was **<u>any</u>** of the **Xanax** (alprazolam) you **misused** in the **<u>past 3 months</u>** prescribed to **<u>YOU</u>**?

1 No, none of it was prescribed to ME
2 Yes, some of it was prescribed to ME
3 Yes, all of it was prescribed to ME
97 Don't know
98 Refuse to answer

99 Staff Only

[If 2m>0]

13. Which of the following **barbiturates** have you **misused** in the **past 3 months**?

Please check all the ones you have misused and hit "next question" to continue

1__ Butisol Sodium (butabarbital)
2__ Nembutal Sodium (pentobarbital)
3__ Seconal Sodium (secobarbital)
4__ Solfoton (phenobarbital)
97 Don't know
98 Refuse to answer
99 Staff Only

[If 13=1]

13a. Was **any** of the **Butisol Sodium** (butabarbital) you **misused** in the past 3 months prescribed to **YOU**?

1 No, none of it was prescribed to ME
2 Yes, some of it was prescribed to ME
3 Yes, all of it was prescribed to ME
97 Don't know
98 Refuse to answer
99 Staff Only

[If 13=2]

13b. Was **any** of the **Nembutal Sodium** (pentobarbital) you **misused** in the past 3 months prescribed to **YOU**?

1 No, none of it was prescribed to ME
2 Yes, some of it was prescribed to ME
3 Yes, all of it was prescribed to ME
97 Don't know
98 Refuse to answer
99 Staff Only

[If 13=3]

13c. Was **any** of the **Seconal Sodium** (secobarbital) you **misused** in the **past 3 months** prescribed to **YOU**?

1 No, none of it was prescribed to ME
2 Yes, some of it was prescribed to ME
3 Yes, all of it was prescribed to ME
97 Don't know
98 Refuse to answer
99 Staff Only

[If 13=4]

13d. Was **any** of the **Solfoton** (phenobarbital) you **misused** in the **past 3 months** prescribed to **YOU**?

1 No, none of it was prescribed to ME
2 Yes, some of it was prescribed to ME
3 Yes, all of it was prescribed to ME
97 Don't know
98 Refuse to answer
99 Staff Only

[If 2n>0]
14. Which of the following have you **misused** in the **past 3 months**?
Please check all the ones you have **misused** and hit "next question" to continue.
1__ methadone
2__ Suboxone (buprenorphine)
97 Don't know
98 Refuse to answer
99 Staff Only

[If 14=1]
14a. Was **any** of the **methadone** you **misused** in the **past 3 months** prescribed to **YOU**?
1 No, none of it was prescribed to ME
2 Yes, some of it was prescribed to ME
3 Yes, all of it was prescribed to ME
97 Don't know
98 Refuse to answer
99 Staff Only

[If 14=2]
14b. Was **any** of the **Suboxone (buprenorphine)** you **misused** in the **past 3 months** prescribed to **YOU**?
1 No, none of it was prescribed to ME
2 Yes, some of it was prescribed to ME
3 Yes, all of it was prescribed to ME
97 Don't know
98 Refuse to answer
99 Staff Only

[If 2o>0]
15. Which of the following **prescription pain killers** have you **misused** in the **past 3 months**? Please check all the ones you have **misused** and hit "next question" to continue.
1__ Darvon (propoxyphene)
2__ Darvocet (propoxyphene)
3__ Demerol (meperidine)
4__ Dilaudid (hydromorphone)
5__ Fentora (fentanyl buccal)
6__ Lortab
7__ OxyContin (oxycodone)

8__ Percocet
9__ Vicodin
97 Don't know
98 Refuse to answer
99 Staff Only

[If 15=1]
15a. Was **<u>any</u>** of the **Darvon** (propoxyphene) you **misused** in the **<u>past 3 months</u>** prescribed to **<u>YOU</u>**?
1 No, none of it was prescribed to ME
2 Yes, some of it was prescribed to ME
3 Yes, all of it was prescribed to ME
97 Don't know
98 Refuse to answer
99 Staff Only

[If 15=2]
15b. Was **<u>any</u>** of the **Darvocet** (propoxyphene) you **misused** in the **<u>past 3 months</u>** prescribed to **<u>YOU</u>**?
1 No, none of it was prescribed to ME
2 Yes, some of it was prescribed to ME
3 Yes, all of it was prescribed to ME
97 Don't know
98 Refuse to answer
99 Staff Only

[If 15=3]
15c. Was **<u>any</u>** of the **Demerol** (meperidine) you **misused** in the **<u>past 3 months</u>** prescribed to **<u>YOU</u>**?
1 No, none of it was prescribed to ME
2 Yes, some of it was prescribed to ME
3 Yes, all of it was prescribed to ME
97 Don't know
98 Refuse to answer
99 Staff Only

[If 15=4]
15d. Was **<u>any</u>** of the **Dilaudid** (hydromorphone) you **misused** in the **<u>past 3 months</u>** prescribed to **<u>YOU</u>**?
1 No, none of it was prescribed to ME
2 Yes, some of it was prescribed to ME
3 Yes, all of it was prescribed to ME
97 Don't know
98 Refuse to answer
99 Staff Only

[If 15=5]

15e. Was **<u>any</u>** of the **Fentora** (fentanyl buccal) you **misused** in the **<u>past 3 months</u>** prescribed to **<u>YOU</u>**?

1 No, none of it was prescribed to ME
2 Yes, some of it was prescribed to ME
3 Yes, all of it was prescribed to ME
97 Don't know
98 Refuse to answer
99 Staff Only

[If 15=6]
15f. Was **<u>any</u>** of the **Lortab** you **misused** in the **<u>past 3 months</u>** prescribed to **<u>YOU</u>**?

1 No, none of it was prescribed to ME
2 Yes, some of it was prescribed to ME
3 Yes, all of it was prescribed to ME
97 Don't know
98 Refuse to answer
99 Staff Only

[If 15=7]
15g. Was **<u>any</u>** of the **OxyContin** (oxycodone) you **misused** in the **<u>past 3 months</u>** prescribed to **<u>YOU</u>**?

1 No, none of it was prescribed to ME
2 Yes, some of it was prescribed to ME
3 Yes, all of it was prescribed to ME
97 Don't know
98 Refuse to answer
99 Staff Only

[If 15=8]
15h. Was **<u>any</u>** of the **Percocet** you **misused** in the **<u>past 3 months</u>** prescribed to **<u>YOU</u>**?

1 No, none of it was prescribed to ME
2 Yes, some of it was prescribed to ME
3 Yes, all of it was prescribed to ME
97 Don't know
98 Refuse to answer
99 Staff Only

[If 15=9]
15i. Was **<u>any</u>** of the **Vicodin** you **misused** in the **<u>past 3 months</u>** prescribed to **<u>YOU</u>**?

1 No, none of it was prescribed to ME
2 Yes, some of it was prescribed to ME
3 Yes, all of it was prescribed to ME
97 Don't know
98 Refuse to answer
99 Staff Only

[Participant Only sees this question if he/she endorsed a drug category that is injectable in life AND if he/she said YES to ever injecting something in the past 3 months]

16. Below are the drugs you said you have used in the **past 3 months.** Please check all the ones you have injected in the **past 3 months** for **non-medical use** and hit next question to continue.

1__ cocaine
2__ crack
3__ crystal meth
4__ ecstasy or MDMA
5__ LSD
6__ PCP
7__ heroin
8__ opium
9__GHB or GBH
10__ Adderall (amphetamine salts)
11__ Concerta (methylphenidate)
12__ Dexedrine (dextroamphetamine)
13__ Focalin (dexmethylphenidate)
14__ Methylin, Metadate or Ritalin (methylphenidate)
15__ Ativan (lorazepam)
16__ Doral (quazepam)
17__ Dalmane (flurazepam)
18__ Halcion (triazolam)
19__ Klonopin (clonazepam)
20__ Librium (chlorodiazepoxide)
21__ Restoril (temazepam)
22__ Tranxene (clorazepate)
23__ Valium (diazepam)
24__ Xanax (alprazolam)
25__ Butisol Sodium (butabarbital)
26__ Nembutal Sodium (pentobarbital)
27__ Seconal Sodium (secobarbital)
28__ Solfoton (phenobarbital)
29__ methadone
30__ Suboxone (buprenorphine)
31__ Darvon (propoxyphene)
32__ Darvocet (propoxyphene)
33__ Demerol (meperidine)
34__ Dilaudid (hydromorphone)
35__ Fentora (fentanyl buccal)
36__ Lortab
37__ OxyContin (oxycodone)
38__ Percocet

39__ Vicodin
97 Don’t know
98 Refuse to answer
99 Staff Only
